# Genome-wide binding analysis of the tomato transcription factor SlDof1 reveals its regulatory impacts on fruit ripening

**DOI:** 10.1186/s43897-021-00011-y

**Published:** 2021-08-28

**Authors:** Yuying Wang, Peiwen Wang, Weihao Wang, Lingxi Kong, Shiping Tian, Guozheng Qin

**Affiliations:** 1 Key Laboratory of Plant Resources, Institute of Botany, Innovation Academy for Seed Design, Chinese Academy of Sciences, Beijing, 100093 China; 2 University of Chinese Academy of Sciences, Beijing, 100049 China

**Keywords:** Tomato, Fruit ripening, Transcriptional regulation, DNA binding with one finger (Dof), chromatin immunoprecipitation followed by sequencing (ChIP-seq), RNA sequencing (RNA-seq)

## Abstract

The DNA binding with one finger (Dof) proteins are plant-specific transcription factors involved in a variety of biological processes. However, little is known about their functions in fruit ripening, a flowering-plant-specific process that is required for seed maturation and dispersal. Here, we found that the tomato Dof transcription factor SlDof1, is necessary for normal fruit ripening. Knockdown of *SlDof1* expression by RNA interference delayed ripening-related processes, including lycopene synthesis and ethylene production. Transcriptome profiling indicated that SlDof1 influences the expression of hundreds of genes, and a chromatin immunoprecipitation sequencing revealed a large number of SlDof1 binding sites. A total of 312 genes were identified as direct targets of SlDof1, among which 162 were negatively regulated by SlDof1 and 150 were positively regulated. The SlDof1 target genes were involved in a variety of metabolic pathways, and follow-up analyses verified that SlDof1 directly regulates some well-known ripening-related genes including *ACS2* and *PG2A* as well as transcriptional repressor genes such as *SlIAA27*. Our findings provide insights into the transcriptional regulatory networks underlying fruit ripening and highlight a gene potentially useful for genetic engineering to control ripening.

**Supplementary Information:**

The online version contains supplementary material available at 10.1186/s43897-021-00011-y.

## Core

The transcription factor SlDof1 is required for normal fruit ripening in tomato. Knockdown of *SlDof1* expression delays ripening-related processes, and transcriptome analysis coupled with ChIP-seq shows that some well-known ripening-related genes are direct targets of SlDof1. Our study demonstrates SlDof1’s regulatory function and provides insights about the molecular regulatory networks controlling fruit ripening.

## Background

Fruits play a major role in seed dispersal and reproductive development in the life cycle of higher plants. Fleshy fruits, which contain many nutrients, including carbohydrates, fibers, vitamins, and antioxidants, are also important components of human food and animal feed. The ripening of fleshy fruits is a complex developmental process that determines the quality of fruits (Giovannoni, 2004). Understanding the molecular mechanisms underlying the regulation of fruit ripening can facilitate the development of new strategies for the improvement of fruit quality and extension of shelf life.

Fruit ripening is tightly controlled by various complex intrinsic signals and environmental factors. Previous research has revealed that the plant hormone ethylene plays a crucial role in the regulation of ripening of climacteric fruits (Barry and Giovannoni, 2006). Recently, research on fruit ripening has been more focused on transcriptional control, which might lie upstream of ethylene signaling and, in some cases, could impact ripening independently of ethylene. Several transcription factors, including RIPENING INHIBITOR (RIN) (Vrebalov et al. 2002), NON-RIPENING (NOR) (Giovannoni et al. 1995; Gao et al. 2020), and COLORLESS NON-RIPENING (CNR) (Manning et al. 2006), were identified as master regulators of fruit ripening in tomatoes (*S. lycopersicum*), although recent studies suggest that a re-evaluation is needed of the function of RIN and NOR in the initiation of ripening (Ito et al. 2017; Li et al. 2018; Gao et al. 2020). Additional tomato transcription factors, such as TOMATO AGAMOUS-LIKE1 (TAGL1) (Itkin et al. 2009; Vrebalov et al. 2009), HD-ZIP HOMEOBOX PROTEIN-1 (HB-1) (Lin et al. 2008), and APETALA2a (AP2a) (Chung et al. 2010; Karlova et al. 2011), were reported to be required for normal ripening in the ethylene-dependent and ethylene-independent modes. Understanding the functional role of transcription factors in fruit ripening will facilitate genetic engineering for the control of ripening and the development of new strategies for the improvement of fruit quality and extension of shelf life.

Chromatin immunoprecipitation followed by microarray hybridization (ChIP-chip) and high-throughput sequencing (ChIP-seq) have emerged as powerful tools to unveil the molecular mechanisms of transcription factor, i.e., by identifying the direct target genes for pivotal transcription factors on a genome-wide scale. Application of these approaches has greatly increased our knowledge regarding key transcription factors involved in plant architecture (Lu et al. 2013), floral development (Yant et al. 2010), defense responses (Birkenbihl et al. 2017), and photomorphogenic development (Zhang et al. 2013). However, genome-wide direct target gene analyses have not been extensively applied to transcription factors involved in fruit ripening. Currently, RIN and FUL1/FUL2 represent the only ripening regulators in tomatoes whose target genes have been identified genome-wide by ChIP-chip or ChIP-seq (Fujisawa et al. 2013; Zhong et al. 2013; Fujisawa et al. 2014). Therefore, although many transcription factors have been revealed to be involved in fruit ripening, our understanding of the transcriptional regulatory networks of ripening is still very limited.

The DNA binding with one finger (Dof) proteins are a plant-specific transcription factor family characterized by a single, highly conserved Dof DNA-binding domain in the N-terminal region (Yanagisawa, 2002). The Dof domain consists of 50–56 amino acid residues, which encompass a C_2_/C_2_ zinc-finger structure, and commonly recognizes the AAAG sequence as a core motif (Gupta et al. 2015). Dof proteins are involved in a wide variety of biological processes in plants, including seed germination (Gabriele et al. 2010), phytochrome signaling (Park et al. 2003), phytohormone responses (Noguero et al. 2015), vascular tissue formation (Konishi and Yanagisawa, 2007), and guard cell development (Negi et al. 2013). Recently, MaDof23 and FaDOF2 were reported to regulate fruit aroma formation in banana fruits (Feng et al. 2016) and strawberries (Molina-Hidalgo et al. 2017), respectively, implying that Dof transcription factors might be associated with fruit ripening processes. In tomatoes, 34 *Dof* genes distributed on 10 chromosomes have been identified based on sequence similarity. These *Dofs* exhibit temporal- and tissue-specific expression patterns (Cai et al. 2013), suggesting their possible regulatory roles in diverse processes. However, the molecular regulatory mechanisms of the Dof family proteins in these processes, such as their target genes, remain elusive. Moreover, the functional roles of Dof proteins in fruit ripening have not been well defined.

In this study, we characterized the function of SlDof1, a tomato Dof transcription factor, in the regulation of fruit ripening. We show that knockdown of *SlDof1* by RNA interference delays ripening-related processes, including lycopene synthesis and ethylene production. Through transcriptome analysis coupled with ChIP-seq, many direct SlDof1 target genes were identified. SlDof1 positively regulated some well-known ripening-related genes including *ACS2* and *PG2A*, and negatively modulated genes encoding transcriptional repressors such as auxin/indole acetic acid (Aux/IAA) proteins.

## Results

### The nucleus-localized SlDof1 protein functions in tomato fruit ripening

**Fig. 1:**
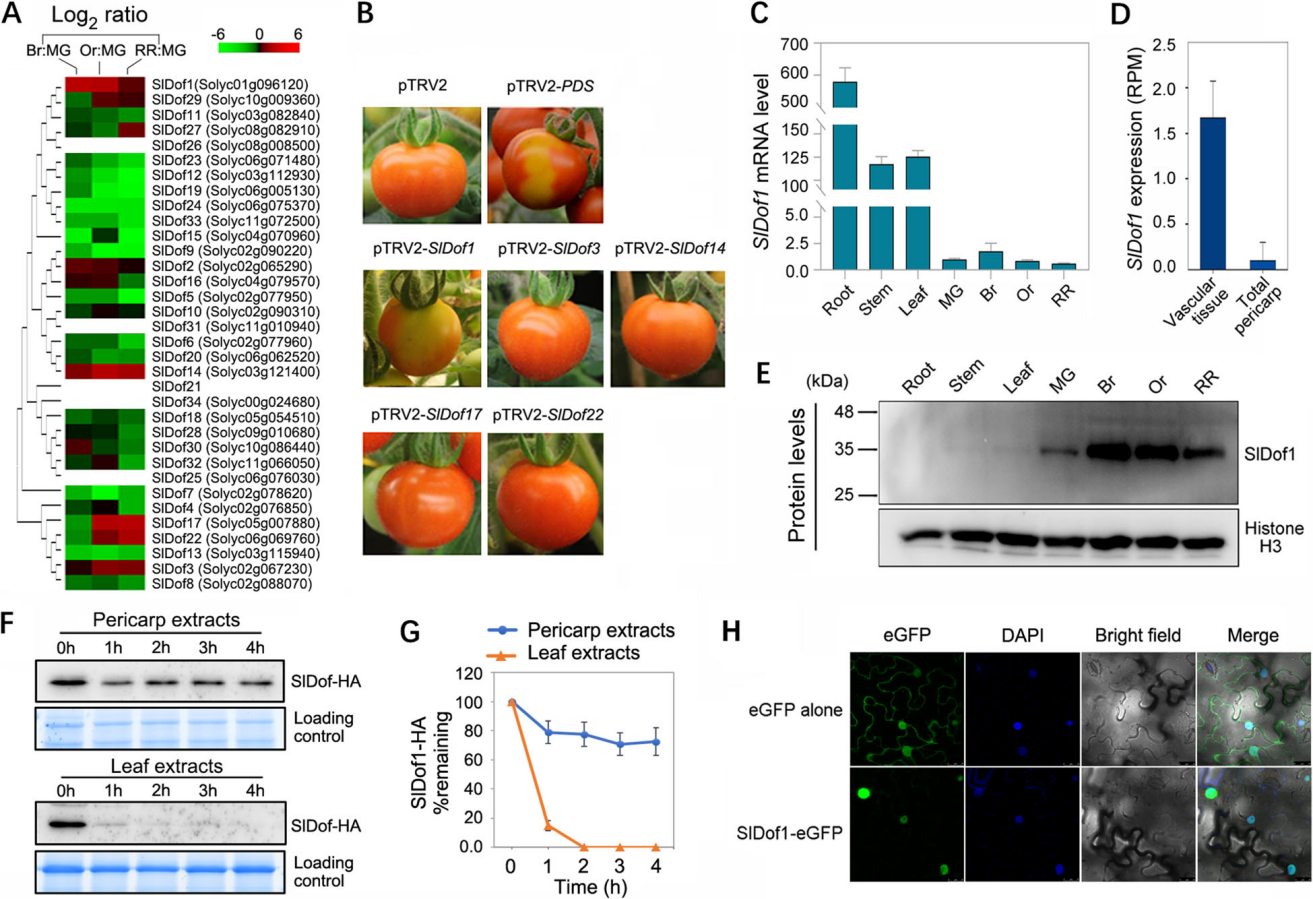
Virus-induced gene silencing (VIGS) screening and expression analysis reveal the involvement of SlDof1 in tomato fruit ripening. (**A**) Phylogenetic analysis of tomato *Dof* genes and expression profiles during fruit ripening, as determined by quantitative RT-PCR. The phylogenetic tree was produced using the neighbor-joining method in MEGA version 6.0 with bootstrapping analysis (1000 replicates). The *ACTIN* gene was utilized as an internal control. The ripening stages include mature green (MG), breaker (Br), orange (Or), and red ripe (RR). Expression ratios were plotted in a heat map on a log_2_ scale, using the MG stage as the denominator. Each row in the color heat map represents a single *Dof* gene, and the gene identifiers (Solyc numbers) are shown. Empty box indicates no expression in fruit. Data from biologically repeated samples are averaged. (**B**) VIGS assay revealing the involvement of *SlDof1* in fruit ripening. Images show the ripe fruit of plants infected with vectors containing no insert (pTRV2; negative control), a specific fragment of *phytoene desaturase* (*PDS*) (pTRV2-*PDS*; positive control), or a specific *Dof* sequence. (**C**) Expression of the *SlDof1* gene in roots, stems, leaves, and pericarps of fruit at different ripening stages, as determined by quantitative RT-PCR. Values are means ± SD of three independent experiments. (**D**) Expression of *SlDof1* in the vascular tissue of the pericarp and the total pericarp based on the Tomato Expression Atlas database (http://tea.solgenomics.net/). RPM, reads per million mapped reads. (**E**) Western blot analysis of SlDof1 protein in the roots, stems, leaves, and pericarps of fruit. An anti-histone H3 immunoblot was used as a protein loading control. (**F**) Cell-free degradation assay of SlDof1. The recombinant SlDof1-HA protein was purified and incubated in extract from tomato pericarps or leaves. The protein levels at different time intervals were measured by immunoblotting using an anti-HA antibody. (**G**) Quantification of protein levels in (**F**) by ImageJ. (**H**) Subcellular localization of SlDof1. *Nicotiana benthamiana* leaves transiently expressing eGFP alone (control) and SlDof1-eGFP were observed under a Leica confocal microscope. The fluorescent dye 4′,6-diamidino-2-phenylindole (DAPI) was used for nuclear staining. Scale bars, 25 μm

Thirty-four Dof transcription factors have been identified in tomatoes, and they show distinct expression patterns in various organs (Cai et al. 2013). However, the function of Dof members in fruit ripening remains unclear. To identify *Dof* genes associated with fruit ripening, we examined the expression patterns of all 34 *Dof* genes at different ripening stages using quantitative RT-PCR analysis. We found that 29 *Dof* genes were expressed during at least one ripening stage (Fig. 1A). Of these, five *Dof* genes (*SlDof1*, *3*, *14*, *17*, and *22*) were upregulated more than two-fold during fruit ripening. These genes were selected for virus-induced gene silencing analysis. The results indicated that only plants silenced for *SlDof1* exhibited an obvious phenotype, fruit color patchiness, suggesting that *SlDof1* participates in the regulation of fruit ripening (Fig. 1B).

The *SlDof1* gene was highly expressed in the roots, stems, and leaves, but expressed at very low levels in the pericarp of fruit (Fig. 1C). Because most of the *Dof* genes are specifically expressed in vascular tissues (Gupta et al. 2015), we compared the expression of the *SlDof1* gene in the vascular tissue of the pericarp with that in the total pericarp using the Tomato Expression Atlas (TEA) database (http://tea.solgenomics.net/; Shinozaki et al. 2018). We found that the *SlDof1* gene was predominantly expressed in the vascular tissue of the pericarp, in which the expression level of *SlDof1* was 16.7-fold higher than that in the total pericarp at the mature green stage (Fig. 1D). This suggested that the extremely low *SlDof1* expression observed in the pericarp of fruit might be caused by the method of sample preparation, in which the mRNA was extracted from the total pericarp.

We then assessed the protein levels of SlDof1 in the vegetative and reproductive tissues. Unexpectedly, the SlDof1 protein specifically accumulated in the pericarp of the fruit, with the highest level at the breaker stage, whereas extremely low levels of SlDof1 proteins, which appeared as blurred bands, were detected in tomato roots, stems, and leaves (Fig. 1E). These data indicated that SlDof1 exhibits notably different expression patterns at the transcriptional and translational levels in tomatoes. To address the discrepancy between the transcript and protein accumulation, we examined SlDof1 protein stability in the vegetative and reproductive tissues using a cell-free degradation assay. The HA-tagged SlDof1 (SlDof1-HA) recombinant protein was purified from *E. coli* and added to total protein extracts prepared from tomato pericarps and leaves. Then, the protein levels were measured at different time intervals by immunoblotting using an anti-HA antibody. Interestingly, the SlDof1 protein was degraded quickly in the protein extracts from leaves, and no SlDof1 protein could be detected after 2 h of incubation (Fig. 1F and G). In contrast, the SlDof1 protein appeared to be more stable in the protein extracts from pericarps and more than 70% of the initial protein remained after 2 h, suggesting that SlDof1 protein stability differs in vegetative and reproductive tissues. These results may partly explain why high levels of the SlDof1 protein were observed in the pericarp of fruit, whereas very low levels accumulated in vegetative tissues.

To examine the cellular localization of SlDof1, the coding sequence of *SlDof1* was cloned into a vector to generate a translational fusion with an enhanced green fluorescent protein (eGFP) at the C-terminus. The construct was introduced into *A. tumefaciens* and then transformed into *Nicotiana benthamiana* leaves. *N. benthamiana* expressing eGFP alone served as a control. Confocal laser scanning microscopy showed that eGFP-tagged SlDof1 (SlDof1-eGFP) produced a specific signal that colocalized with DAPI-stained nuclei, whereas the eGFP-alone control produced a fluorescent signal that was observed throughout the cell (Fig. 1H). This indicated that SlDof1 is specifically located in the nucleus.

### Knockdown of *SlDof1* delays fruit ripening

**Fig. 2:**
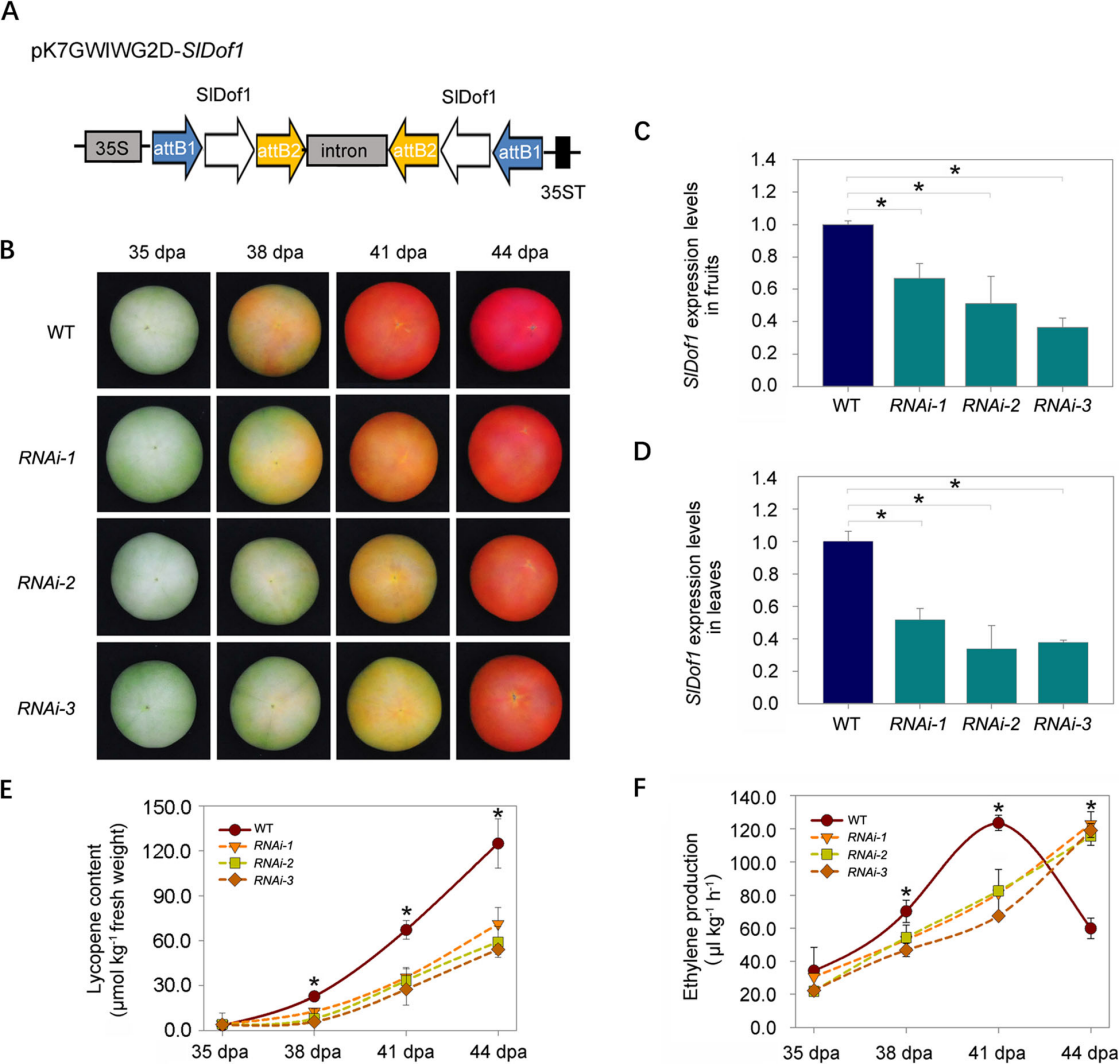
*SlDof1* is required for normal tomato fruit ripening. (**A**) Diagram depicting the recombinant RNAi vector used in this study. The specific *SlDof1* fragment was cloned into pK7GWIWG2D to generate the plasmid pK7GWIWG2D-*SlDof1*. The *SlDof1* RNAi plasmid was transformed into tomatoes using the *A. tumefaciens*-mediated transformation method. 35S, CaMV 35S promoter; attB1 and attB2, Gateway recombination sites; 35ST, CaMV 35S terminator. (**B**) Ripening phenotype of *SlDof1* RNAi lines. Fruits from wild type (WT) and *SlDof1* RNAi lines (*RNAi-1*, *RNAi-2*, and *RNAi-3*) at 35 days post-anthesis (dpa), 38 dpa, 41 dpa, and 44 dpa are shown. (**C**) Expression of *SlDof1* in the fruit of the WT and *SlDof1* RNAi lines as determined by quantitative RT-PCR. (**D**) Expression of *SlDof1* in leaves of the WT and *SlDof1* RNAi lines. In (**C**) and (**D**), the gene transcript levels were normalized against *ACTIN*, followed by normalization against WT expression. (**E**) Lycopene accumulation in the WT and *SlDof1* RNAi fruit during ripening. (**F**) Ethylene production in the WT and *SlDof1* RNAi fruit during ripening. In (**C**) to (**F**), values are expressed as the means ± SD of three replicates. Asterisks indicate significant differences (*P* < 0.05; Student’s *t*-test) between WT and *SlDof1* RNAi lines

To gain insights into the function of *SlDof1*, we generated a construct expressing *SlDof1* RNAi under the control of the cauliflower mosaic virus (CaMV) 35S promoter (Fig. 2A). The construct was transformed into the wild-type tomato cultivar Ailsa Craig. Three independent RNAi lines (*RNAi-1*, *RNAi-2*, and *RNAi-3*) with confirmed transgene integration presented obvious and similar ripening-related phenotypes (Fig. 2B). The differences in fruit ripening between the *SlDof1* RNAi lines and the wild type appeared to be distinct at 38 days post-anthesis (dpa). A visible change in color occurred at this stage in the wild-type fruit, whereas the *SlDof1* RNAi tomatoes were almost green. At 41 dpa, the wild-type fruit was a homogenous orange color, whereas the fruits from the *SlDof1* RNAi lines were only just beginning to change color. Notably, the fruits of *SlDof1* RNAi plants eventually fully ripened, suggesting that *SlDof1* only partly influenced fruit ripening. Alternatively, homologous genes may exist that complement the function of *SlDof1*. We did not find any phenotypes related to roots, stems, or leaves, even though the *SlDof1* gene was preferentially expressed in vegetative organs. This could be explained by functional redundancy caused by the activity of other *Dof* genes.

To determine whether *SlDof1* was specifically repressed in the RNAi lines, total RNA from fruits and leaves of the wild-type and transgenic lines was extracted and used as template for quantitative RT-PCR. The transcript levels of *SlDof1* were significantly lower in both the fruits and leaves of transgenic plants compared with those of the wild type (Fig. 2C, D). Using the computational tool pssRNAit (http://plantgrn.noble.org/pssRNAit), no potential off-targets for the RNAi construct were identified, indicating that the RNAi construct was specific for *SlDof1*. We also examined the expression of *SlDof29* and *SlDof11*, which are closely related to *SlDof1*, and the expression of *SlDof8*, *SlDof14*, *SlDof30*, and *SlDof32*, which exhibit high expression in tomato fruits. The mRNA levels of these genes were not significantly altered in any of the three RNAi lines (*RNAi-1*, *RNAi-2*, and *RNAi-3*) compared with those in the wild type (Fig. S1). These results demonstrated the specificity of the *SlDof1* RNAi construct for the target gene. The three lines (*RNAi-1*, *RNAi-2*, and *RNAi-3*) were selected for further analysis.

Because color changes in ripe fruit were observed between the wild type and *SlDof1* RNAi lines, we measured the lycopene content to determine the underlying causes. The contents of lycopene in fruit from the *SlDof1* RNAi lines were less than 50% of the wild-type level at 41 dpa and less than 60% at 44 dpa (Fig. 2E), indicating that *SlDof1* expression influenced lycopene accumulation during fruit ripening.

As a climacteric fruit, the ripening of tomatoes requires an increase in ethylene biosynthesis (Barry and Giovannoni, 2006). We evaluated whether the delay in fruit ripening in the *SlDof1* RNAi lines was associated with the production of ethylene. As shown in Fig. 2F, fruits from the transgenic lines (*RNAi-1*, *RNAi-2*, and *RNAi-3*) generated less ethylene than did those from the wild type at 38 dpa and 41 dpa but more ethylene at 44 dpa, indicating a delay in the generation of the climacteric ethylene peak; this may have contributed to the delay in fruit ripening.

### SlDof1 affects the expression of a large number of genes

**Fig. 3:**
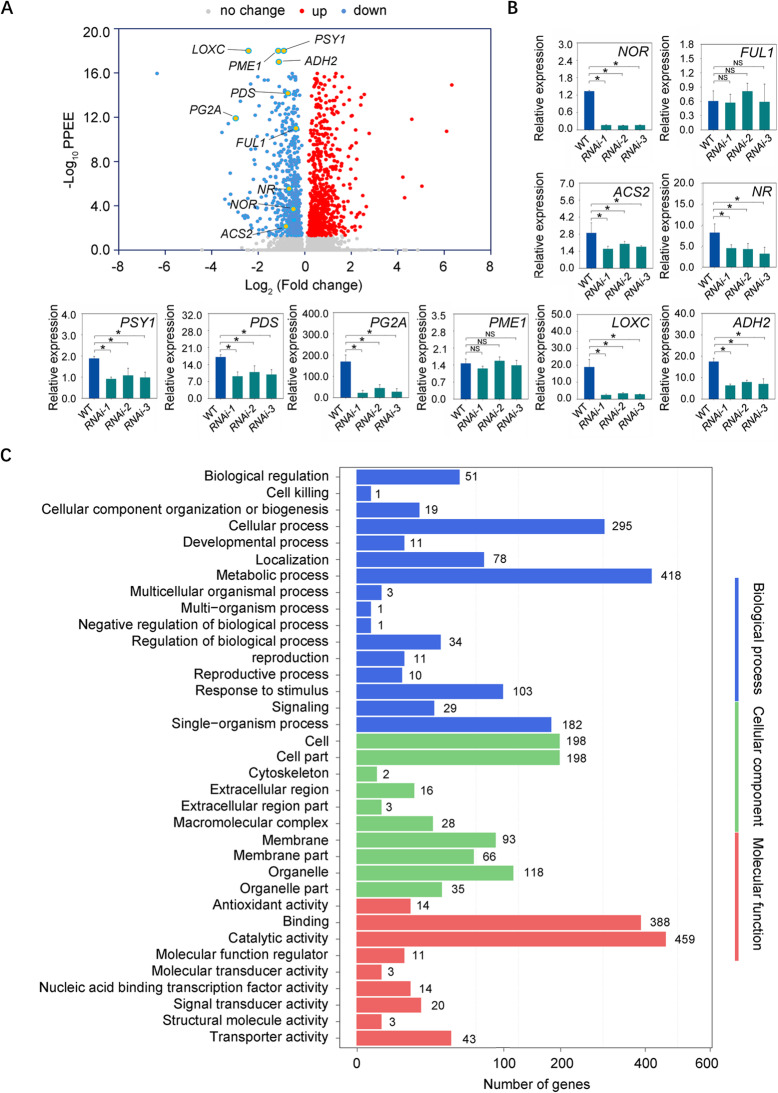
SlDof1 affects the expression of hundreds of genes as revealed by RNA sequencing. (**A**) Volcano plot visualization of RNA sequencing data. Red points represent upregulated genes, and blue points represent downregulated genes, in *SlDof1* RNAi fruit compared with that of the wild type at 38 days post-anthesis. Ten ripening-related genes are indicated by orange points. (**B**) Quantitative RT-PCR validation of the relative expression levels of the 10 ripening-related genes in the fruit of the wild-type (WT) and *SlDof1* RNAi lines. Values are means ± SD of three biological replicates. Asterisks indicate significant differences (*P* < 0.05; Student’s *t*-test). NS, not significant. *NOR*, *nonripening*; *FUL1*, *fruitfull 1*; *ACS2*, *ACC synthase 2*; *NR*, *never-ripe*; *PSY1*, *phytoene synthase 1*; *PDS*, *phytoene desaturase*; *PG2A*, *polygalacturonase A*; *PME1*, *pectinesterase 1*; *LOXC, lipoxygenase C*; *ADH2*, *alcohol dehydrogenase 2*. (**C**) Gene Ontology (GO) analysis of genes that were differentially expressed in the *SlDof1* RNAi fruit compared with that of the wild type. The number of genes belonging to each GO category is shown

To identify genes affected by SlDof1 during fruit ripening, we performed comparative transcriptome sequencing (RNA-seq) of wild-type and *SlDof1* RNAi fruit at 38 dpa with three biological replicates. The numbers of total reads and mapped reads and the ratio of mapped reads in each replicate are shown in Fig. S2A. The reads from three biological replicates for each sample were highly correlated based on Pearson’s correlation coefficient, indicating the high reproducibility and reliability of the RNA-seq data (Fig. S2B). A total of 1728 genes were differentially expressed in the *SlDof1* RNAi fruit compared with the wild-type fruit (Fig. 3A and Data S1). Among these genes, 872 (50.5%) were upregulated in the *SlDof1* RNAi fruit and 856 (49.5%) were downregulated (Fig. 3A and Data S1). We successfully identified multiple ripening-related genes, including *pectinesterase 1* (*PME1*), *PME2*, and *polygalacturonase 2A* (*PG2A*), which are involved in cell wall degradation; *ACC synthase 2* (*ACS2*), *never-ripe* (*NR*), and *ethylene insensitive 3* (*EIL*), which are associated with ethylene biosynthesis and signaling; *lipoxygenase A* (*LOXA*), *LOXB*, *LOXC*, and *alcohol dehydrogenase 2* (*ADH2*), which are related to aroma formation; and *phytoene synthase 1* (*PSY1*) and *phytoene desaturase* (*PDS*), which are involved in carotenoid biosynthesis (Data S1). All of these genes were downregulated in the *SlDof1* RNAi fruit. We selected 10 of these ripening-related genes (*NOR*, *FUL1*, *ACS2*, *NR*, *PSY1*, *PDS*, *PG2A*, *PME1*, *LOXC*, and *ADH2*) for quantitative RT-PCR analysis to validate the results of RNA-seq. As shown in Fig. 3A and B, most of these genes were downregulated in the *SlDof1* RNAi fruit, which was highly correlated with that in the RNA-seq data. Gene Ontology (GO) analysis of the differentially expressed genes revealed potential functions of SlDof1 in three categories, namely biological processes, molecular functions, and cellular components (Fig. 3C). “Metabolic process” appeared to be the most highly represented biological process term, and 418 genes with differential expression were annotated with this term. The major cellular component term was “cell part”, among which 198 differentially expressed genes were annotated to this term. The most significantly enriched molecular function term was “catalytic activity”, which included 459 genes with differential expression.

### Genome-wide identification of SlDof1 binding regions in tomato fruits

**Fig. 4:**
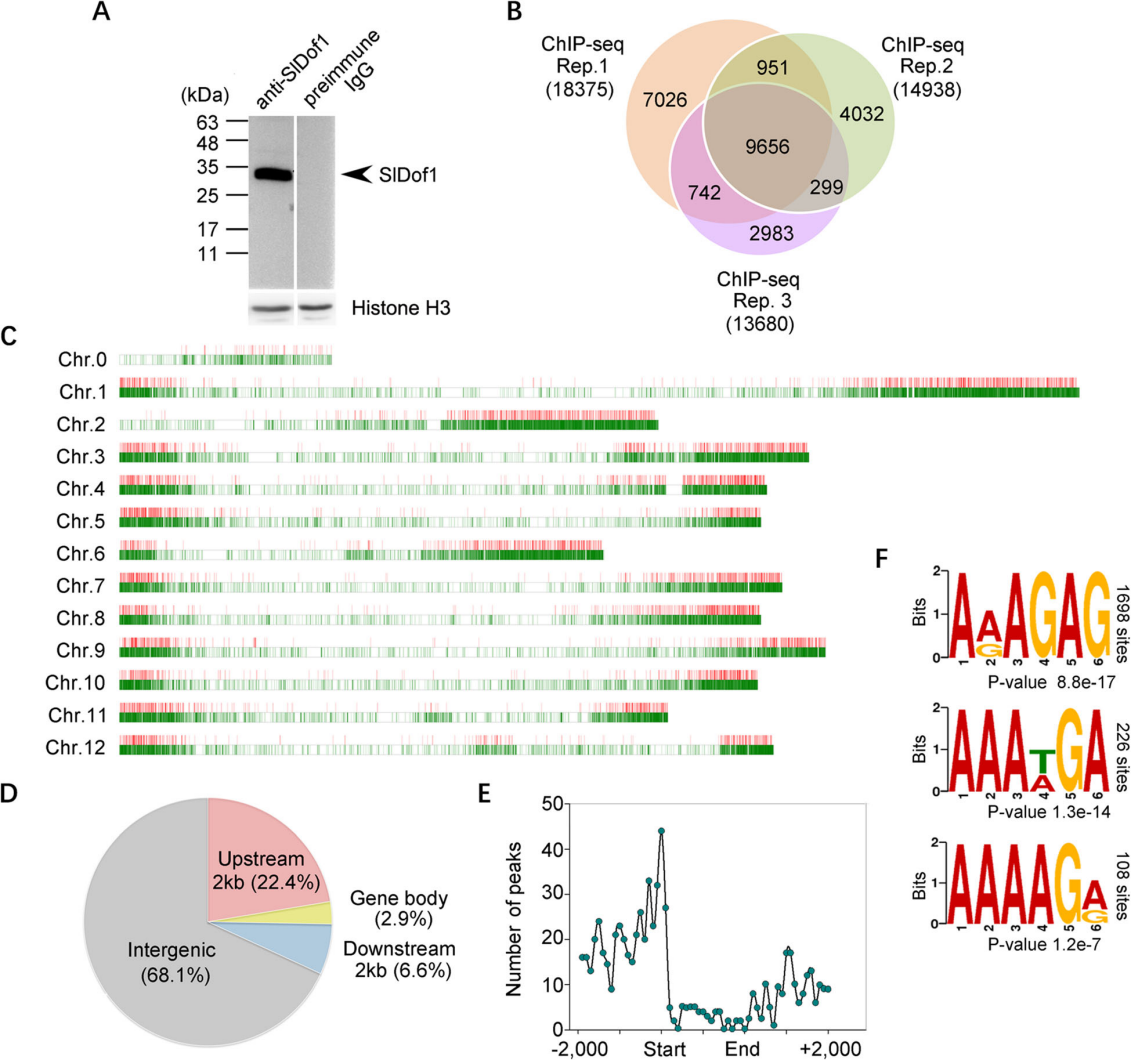
Chromatin immunoprecipitation sequencing reveals SlDof1 binding sites in tomato fruit. (**A**) Western blot analysis of the affinity-purified SlDof1 polyclonal antibody used for the chromatin immunoprecipitation assay. Nuclear proteins from the wild-type fruit at 38 days post-anthesis were hybridized with the purified SlDof1 antibody or preimmune serum. (**B**) Venn diagram depicting the numbers of SlDof1 binding peaks in three biological replicates of chromatin immunoprecipitation sequencing (ChIP-seq). Peaks were obtained by the MACS program with a *P*-value cut-off of 1E-5. (**C**) Genome-scale view of SlDof1 binding sites across the 12 chromosomes of tomato. The SlDof1 target genes are depicted as red bars, whereas normally expressed genes are depicted as green bars. (**D**) Distribution of SlDof1 binding peaks across genomic features. Peaks found within 2 kb upstream of a transcription start site and downstream of a translation termination site were categorized as upstream and downstream sites, respectively. Peaks that were found further upstream or downstream were categorized as intergenic sites. (**E**) Distribution of SlDof1 binding peaks relative to the genic region. SlDof1 binding sites are highly enriched in the region proximal to the transcriptional start site. (**F**) Binding motifs identified in the SlDof1 binding peaks. The top three motifs identified using the DREME software are shown

To further understand the regulatory mechanisms of SlDof1 in fruit ripening, the genome-wide DNA-binding sites of SlDof1 were investigated using a ChIP-seq approach. A polyclonal antibody against SlDof1 was raised and affinity purified. Immunoblot analysis indicated that the purified SlDof1 antibody reacted exclusively with the SlDof1 protein. A signal detected by the affinity-purified anti-SlDof1 antibody corresponded to the size (33 kDa) of the predicted full-length SlDof1 protein (Fig. 4A, arrowhead), but no immunoreactive bands were detected when preimmune serum was used on extracts from wild-type tomato fruit at 38 dpa. For the ChIP-seq assay, SlDof1-bound chromatin from tomato pericarps at 38 dpa was immunoprecipitated with the SlDof1-specific antibody. To ensure the reliability of the data, three biologically independent immunoprecipitated samples were employed for library construction and high-throughput sequencing (Fig. S3).

Using Model-based Analysis for ChIP-seq (MACS), a total of 18,375, 14,938, and 13,680 peaks were obtained from the three biological replicates (Fig. 4B and Data S2). Comparative analysis of these peaks revealed 9656 overlapping peaks, which were considered high-confidence SlDof1-binding regions and were used for further analysis (Fig. 4B and Data S2). Genome-wide distribution analysis indicated that the DNA-binding sites of SlDof1 were distributed throughout the tomato genome but were rare in the centromere regions (Fig. 4C). SlDof1 bound to various genomic segments, including the promoter region (2 kb upstream from the transcription start site), gene body, 3′ untranslated region (2 kb downstream from the translation termination site), and intergenic region. Of these SlDof1 binding sites, 22.4% were located in promoter regions (Fig. 4D). Detailed analysis of the SlDof1 binding profile in promoter regions revealed that the peak was close to the transcription start site, i.e., − 200 to + 100 bp relative to the transcription start site (Fig. 4E). This distribution pattern was consistent with SlDof1 being a typical transcription factor with DNA-binding ability and gene regulatory activity.

To gain more insight into the DNA-binding properties of SlDof1 in fruit ripening, de novo motif prediction was conducted based on the SlDof1 binding regions identified in our ChIP-seq data using the Discriminative Regular Expression Motif Elicitation (DREME) tools. Three abundant motifs were identified by this method. The most enriched motif was represented by A(A/G) AGAG, which accounted for 1698 SlDof1 binding sites (Fig. 4F). The second, AAA(T/A) GA, and third, AAAAG(A/G), most highly enriched motifs were present in 226 and 108 SlDof1-binding sites, respectively (Fig. 4F). These DNA-binding sequences differed slightly from the previously reported Dof recognition motif (AAAG). It has been shown that, besides the canonical binding motif, Dof transcription factors can recognize other sequences, such as the (A/T) AAAG sequence or AGTA motif (Kisu et al. 1998; Yanagisawa and Schmidt, 1999). This suggests that the precise selection of target gene promoter sequences by each Dof protein in vivo could require other factors in addition to the DNA sequence.

### Identification of SlDof1 direct target genes

**Fig. 5:**
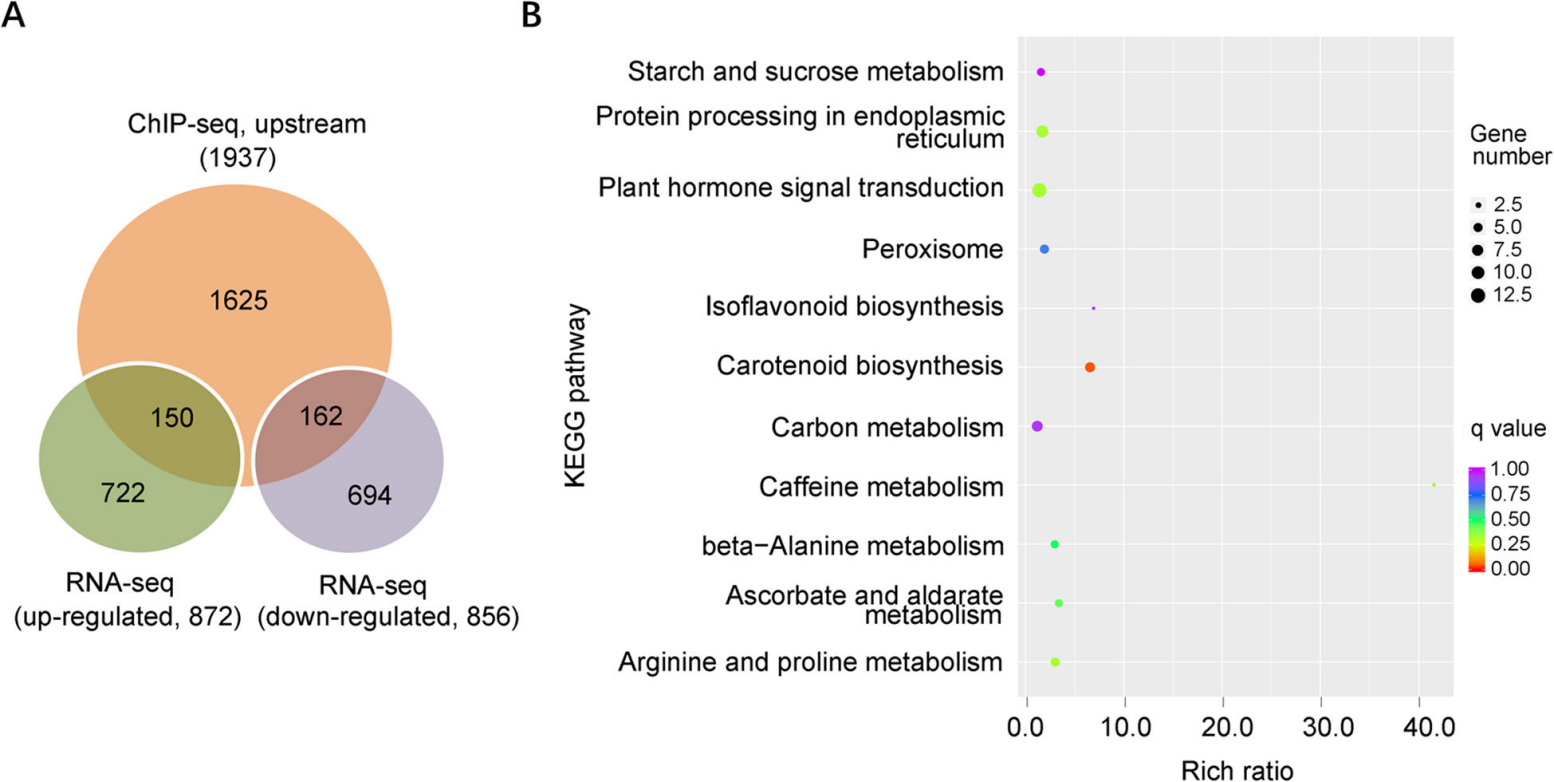
Genome-wide identification of SlDof1 direct target genes. (**A**) Venn diagram showing the overlap between genes bound by SlDof1 from chromatin immunoprecipitation sequencing (ChIP-seq) and genes differentially expressed in *SlDof1* RNAi fruit determined by RNA sequencing (RNA-seq). (**B**) Kyoto Encyclopedia of Genes and Genomes (KEGG) analysis of SlDof1 target genes. The size of the circle represents the number of genes belonging to that KEGG pathway, and the color represents the q value of pathway enrichment determined by Fisher’s exact test with Bonferroni correction

As a transcription factor, SlDof1 regulates gene expression by binding to promoter regions. Therefore, to identify the direct target genes of SlDof1, we focused on the ChIP-seq peaks that were located within the 2 kb upstream region starting from the transcription start site. A total of 2161 peaks corresponding to 1937 genes were identified (Data S3) considered to be potential SlDof1 direct targets. The RNA-seq and ChIP-seq data were merged and 312 (16.1%) overlapping genes were revealed (Fig. 5A). These genes were recognized as SlDof1 direct targets. Among them, 162 genes (51.9%) were upregulated in the *SlDof1* RNAi tomatoes and could be considered negatively regulated SlDof1 targets. In contrast, 150 genes (48.1%) were downregulated after *SlDof1* was repressed, and represent positively regulated SlDof1 targets (Fig. 5A and Data S4). The data obtained here indicated that SlDof1 functions both as a transcriptional repressor and an activator during fruit ripening. Transcription factor RIN has also been shown to regulate gene expression as both an activator and a repressor during fruit ripening in tomatoes (Fujisawa et al. 2013).

Kyoto Encyclopedia of Genes and Genomes (KEGG) analysis showed that SlDof1 target genes were involved in multiple metabolic pathways, including starch and sucrose metabolism, plant hormone signal transduction, and carotenoid biosynthesis (Fig. 5B). Multiple genes involved in plant hormone signal transduction were identified in the SlDof1-binding gene set, suggesting that one of the major functions of SlDof1 might be direct modulation of plant signaling components.

### Multiple ripening-related genes are direct targets of SlDof1

**Fig. 6:**
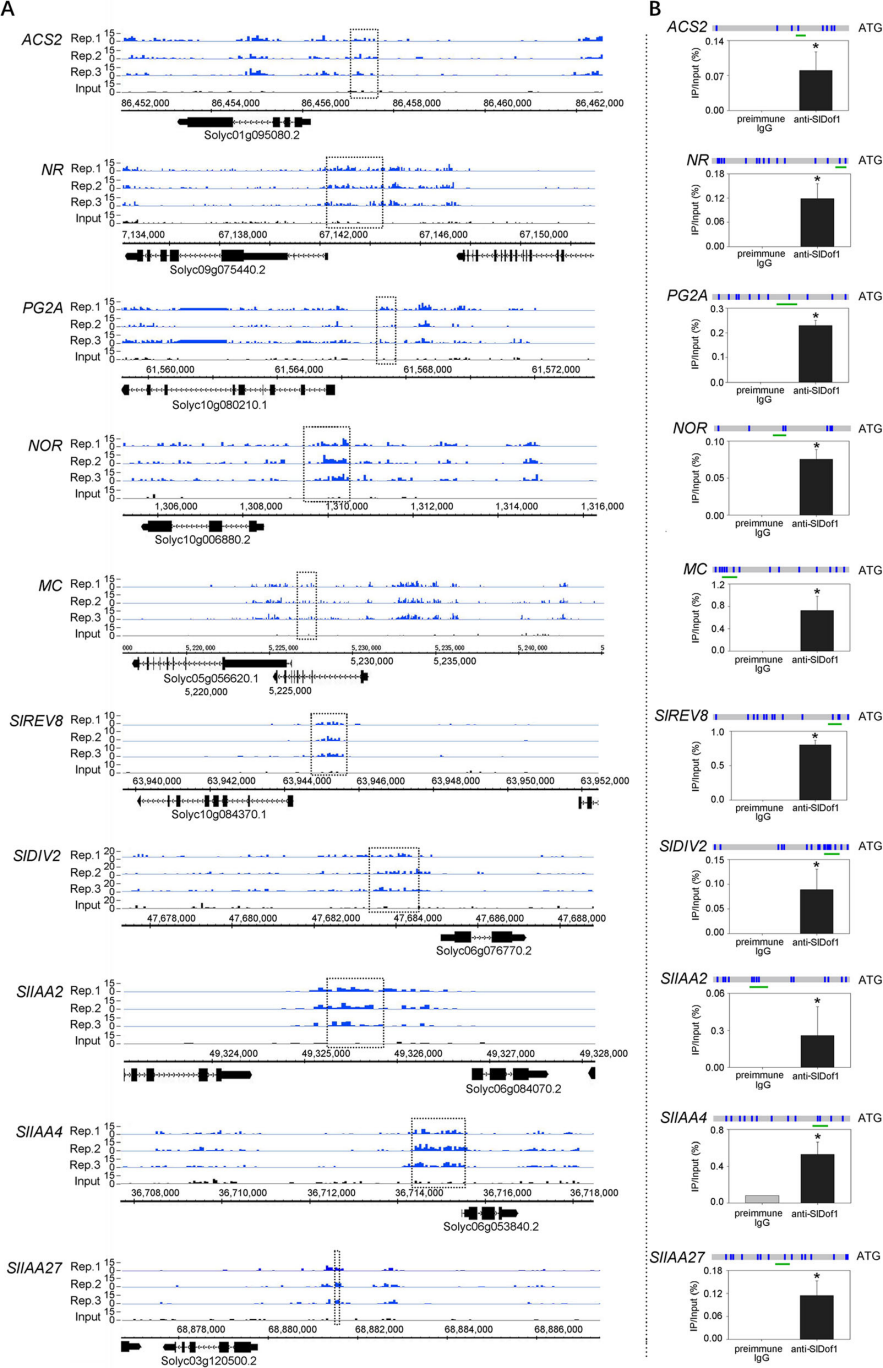
Validation of SlDof1 direct target genes by ChIP-qPCR. (**A**) The raw chromatin immunoprecipitation sequencing peaks from three biological replicates for indicated genes are shown. Black dot-line rectangles mark the peak regions that were detected within 2 kb upstream of the transcription start site in all three biological replicates. The structure of the corresponding gene is presented below with black bars representing exons and lines representing introns. The direction of transcription is indicated by arrows. (**B**) ChIP-qPCR analysis reveals that SlDof1 binds to the promoter regions of selected genes. The promoter structures of the target genes are shown. Blue boxes represent SlDof1 binding motifs. Green lines indicate the region used for ChIP-qPCR. All primers were designed based on the peak sequences enriched in ChIP sequencing. Values are the percentage of DNA fragments that co-immunoprecipitated with anti-SlDof1 antibodies or non-specific antibodies (preimmune IgG) relative to the input DNAs. Error bars represent the SD of three independent experiments. Asterisks indicate significant differences (*P* < 0.05; Student’s *t*-test). *ACS2*, *ACC synthase 2*; *NR*, *never-ripe*; *PG2A*, *polygalacturonase A*; *NOR*, *nonripening*; *MC*, *macrocalyx*; *SlREV8*, a homolog of *Arabidopsis REVEILLE8*; *SlDIV2*, a homolog of *Arabidopsis DIVARICATA*; *SlIAA2*, *Aux/IAA 2*; *SlIAA4*, *Aux/IAA 4*; *SlIAA27*, *Aux/IAA 27*

To verify that SlDof1 binds to the sites detected by ChIP-seq and to determine how SlDof1 regulates fruit ripening, we performed ChIP-qPCR assays. Several well-known ripening-related genes and regulators from the list of putative SlDof1 targets (Data S4), which were revealed by ChIP-seq and showed differential expression in the *SlDof1* RNAi fruits, were chosen for analysis. As shown in Fig. 6A, B, SlDof1 bound to the promoters of two genes (*ACS2* and *NR*) relevant to ethylene synthesis and signaling, suggesting that SlDof1 participates in the ethylene signaling pathway. In addition, the ability of SlDof1 to bind to the promoter regions of genes associated with cell wall metabolism, e.g., *PG2A*, and a gene encoding the global ripening regulator *NOR* were validated by ChIP-qPCR. Besides these well-known ripening-related genes, SlDof1 also bound to the promoters of three genes, *SlIAA2*, *SlIAA4*, and *SlIAA27*, encoding Aux/IAA proteins (Fig. 6A, B), which contain potent transcriptional repression domains and can repress transcription of auxin-responsive genes (Tiwari et al. 2004). Furthermore, SlDof1 bound to the promoters of several transcription factor genes such as *macrocalyx* (*MC*) and two *MYB-like* genes, one of which (*SlREV8*, Solyc10g084370) is a homolog of *Arabidopsis REVEILLE8* and another one (*SlDIV2*, Solyc06g076770) is a homolog of *Arabidopsis DIVARICATA* (Fig. 6A, B).

Notably, among these SlDof1 target genes, the well-known ripening-related genes (*ACS2*, *NR*, *PG2A*, and *NOR*) were down-regulated in the *SlDof1* RNAi fruits (Fig. 3A, B and Data S1), indicating that they are positively regulated direct targets of SlDof1. In contrast, *MC*, *MYBs*, and two *Aux/IAA* genes were upregulated in *SlDof1* RNAi fruit, demonstrating that they are negatively regulated direct targets of SlDof1. We also investigated the ability of SlDof1 to bind to other well-characterized ripening-related genes, such as *PSY1* and *ADH2*, which were identified as overlapping genes in RNA-seq and ChIP-seq, but no enrichment was observed (Fig. S4), indicating that they are indirectly regulated by SlDof1 during fruit ripening.

### SlDof1 functions as in both transcriptional activation and repression

**Fig. 7:**
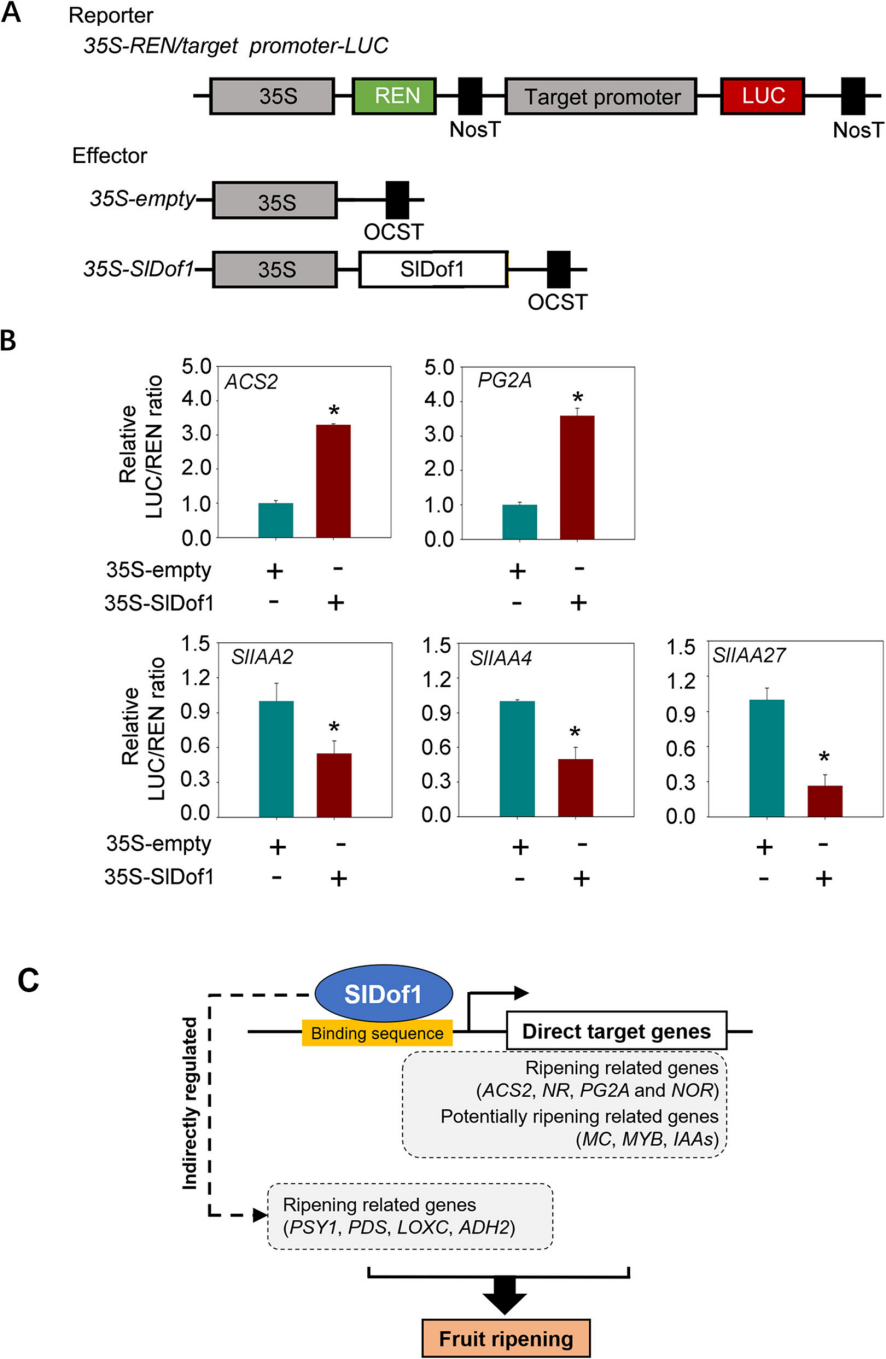
Transcriptional activity assay of SlDof1 in *Nicotiana benthamiana* leaves. (**A**) Diagram depicting the construction of the reporter and effector plasmids used in this assay. The reporter plasmids contain the promoters of putative SlDof1 target genes fused with *firefly luciferase* (*LUC*), with *renilla luciferase* (*REN*) driven by the CaMV35S promoter as an internal control. The *SlDof1* coding sequence was cloned into the effector plasmid under the control of CaMV35S. The empty effector vector (35S-empty) was used as a negative control. The reporters and effectors were co-transformed into *N. benthamiana* leaves using the *A. tumefaciens*-mediated transformation method. OCST, OCS terminator. (**B**) Transcription activation or repression activity, which is expressed by the ratio of LUC to REN. Values are means ± SD of six independent experiments. Asterisks indicate statistically significant differences (*P* < 0.05; Student’s *t*-test). (**C**) A schematic summary of the mechanism by which SlDof1 regulates fruit ripening

To examine the transcriptional activity of SlDof1 on these potential target genes, we performed transient transcription assays in *N. benthamiana* using a dual reporter system. The dual reporter vector contained the promoter regions of the putative SlDof1 target genes fused with the *firefly luciferase* (*LUC*) reporter gene, with *renilla luciferase* (*REN*) driven by CaMV35S as an internal control; the effector vector contained the *SlDof1* coding sequence driven by CaMV35S (Fig. 7A). The constructs were co-infiltrated into *N. benthamiana* leaves using *A. tumefaciens*. As shown in Fig. 7B, SlDof1 activated the expression of *ACS2* and *PG2A*, which was indicated by an increase in the LUC to REN ratio in *N. benthamiana* expressing SlDof1 compared with that in plants expressing the negative control (empty effector vector). In contrast, SlDof1 repressed the expression of *SlIAA2*, *SlIAA4*, and *SlIAA27*, which was indicated by a decrease in the LUC to REN ratio in *N. benthamiana* expressing SlDof1 compared with that in plants expressing the negative control. These results confirmed that SlDof1 functions both as a transcriptional activator and repressor. Notably, the transcriptional activity of SlDof1 on *NR*, *NOR*, *MC*, *SlDIV2*, and *SlREV8* was not detected. It remains possible that the transient transcription assay conducted in *N. benthamiana* leaves does not completely reflect the regulatory activity of SlDof1 in fruits, which are more complicated than *N. benthamiana* leaves.

## Discussion

### SlDof1 positively regulates multiple ripening-related genes

In this study, several well-known ripening-related genes, including *ACS2*, *NR*, *NOR*, and *PG2A*, were identified as direct targets of SlDof1 (Fig. 6 and Data S4). *ACS2* and *NR* are critical for the ethylene signaling pathway in tomatoes. *ACS2* encodes one of the key enzymes for ethylene biosynthesis and silencing of *ACS2* causes a delay in fruit ripening with strong inhibition of ethylene production (Hamilton et al. 1990). *NR* represents one of the most important components of the ethylene receptor family in ripe fruit tissue. A mutation in tomato *NR* causes an unripe fruit phenotype (Wilkinson et al. 1995). The identification of *ACS2* and *NR* as direct targets indicates that SlDof1 participates in the regulation of ethylene production and response during fruit ripening.

*NOR* is a *NAC* gene encoding one of the global regulators of fruit ripening, and a mutation in *NOR* results in ripening inhibition in tomatoes (Giovannoni, 2004; Gao et al. 2020). The expression of *NOR* was reduced in the *SlDof1* RNAi fruit, suggesting that *NOR* is positively regulated by SlDof1. Considering that RIN acts downstream of NOR (Fujisawa et al. 2013), it is expected that *RIN* expression will be decreased in *SlDof1* RNAi fruit. However, our RNA-seq data indicated that the expression of *RIN* was not significantly altered in the *SlDof1* RNAi fruit. This could be explained by the partial regulation of *NOR* by SlDof1; a partial decrease in *NOR* expression may not lead to a change in *RIN* expression. Also, the regulation of *RIN* by SlDof1 may occur at other ripening stages (such as Or and RR) other than the stage when the samples were harvested for RNA-seq. To further understand the role of SlDof1 in the regulation network of fruit ripening, we examined whether *SlDof1* is regulated by RIN by analyzing the expression of *SlDof1* in *rin* mutant fruit. Quantitative RT-PCR showed that the mRNA levels of *SlDof1* were significantly reduced in *rin* mutant fruit (Fig. S5A). However, a ChIP-qPCR assay indicated that RIN could not bind to the promoter of *SlDof1* (Fig. S5B), suggesting that RIN regulates *SlDof1* indirectly.

The identification of *PG2A* as a direct target of SlDof1 indicated that SlDof1 is involved in cell wall metabolism, and this is consistent with previous studies that demonstrated that Dof proteins could regulate multiple genes associated with cell wall metabolism, including *PG* (Wei et al. 2010), *XET* (Xu et al. 2016), and *MaEXP1/2/3/5* and *MaPME3* (Feng et al. 2016).

### SlDof1 negatively regulates several transcription factors

An interesting finding of this study was that, besides NOR, several other transcription factors, including MC and MYB transcription factors, were identified as direct targets of SlDof1 (Fig. 6 and Data S4). *MC* exhibits substantial similarity to *Arabidopsis APETALA1* (*AP1*), which is a class A MADS-box gene. Antisense *MC* expression led to indeterminate inflorescences with large sepals (Vrebalov et al. 2002), indicating that *MC* is required for tomato inflorescence determinacy and sepal development. Recent studies have demonstrated that MC contains an ethylene response factor (ERF)-associated amphiphilic repression (EAR) motif-like sequence, which displays clear transcriptional repressor activity (Ito et al. 2017; Li et al. 2018). Furthermore, the RIN-MC chimeric protein, which is generated by translation of the in-frame fusion of the adjacent truncated *RIN* and *MC* coding sequences in the *rin* mutant, could directly bind the promoters of ripening-related genes, such as *ACS4*, *PSY1*, *PG2A*, *PL*, *RIN*, and *CNR* and repress their expression (Ito et al. 2017). This suggests that the EAR motif-like sequence in MC might exhibit repression activity toward ripening-related genes. In this study, *MC* was directly regulated by SlDof1. We speculate that SlDof1 might regulate fruit ripening by negatively regulating *MC*. Further research is needed to determine the possible function of MC in fruit ripening and its regulatory mechanisms.

MYB transcription factors are involved in the regulation of multiple plant-specific processes, including plant development, secondary metabolism, and environmental stress responses (Ambawat et al. 2013). Some MYBs act as transcriptional repressors during normal plant development, such as AtMYB32 in pollen development (Preston et al. 2004) and AtMYB2 in flavonol accumulation (Dubos et al. 2008). In the present study, several *MYBs* were identified as being negatively regulated by SlDof1 (Fig. 6 and Data S4). Whether these *MYB* genes are necessary for fruit ripening is currently unknown, and elucidating their functions would help to clarify the mechanisms of SlDof1.

### *Aux/IAA* genes in the auxin signaling pathway are regulated by SlDof1

Plant hormones play critical roles in fruit ripening. In addition to ethylene, which has been extensively studied in climacteric fruits, auxin has been reported to be associated with fruit ripening because the exogenous application of auxin delays the ripening process in tomatoes (Vendrell 1985). Auxin might participate in the ripening process directly by controlling the expression of auxin-responsive genes or indirectly through regulation of ethylene biosynthesis and signaling (Trainotti et al. 2007; Liu et al. 2018). Two types of transcription factor families, the auxin response factor (ARF) and Aux/IAA proteins, are required for transcriptional regulation of auxin-responsive genes. ARFs act as transcriptional activators that reside on auxin-responsive promoter elements, whereas Aux/IAA proteins, which contain an active repression domain resembling the EAR motif found in some ERFs (Tiwari et al. 2004), appear to function as transcriptional repressors by interacting with ARFs. Recent studies have demonstrated that SlARF2 and SlARF4 participate in the regulation of fruit ripening in tomatoes (Sagar et al. 2013; Hao et al. 2015). Tomatoes contain 25 *Aux/IAA* genes (Audran-Delalande et al. 2012), among which *SlIAA9* and *SlIAA27* have been reported to be involved in fruit set and development (Wang et al. 2005; Bassa et al. 2012), but the regulation of these *Aux/IAAs* remains largely unknown.

In the present study, we found that SlDof1 bound directly to the promoters of three *Aux/IAA* genes (*SlIAA2*, *SlIAA4*, and *SlIAA27*) and regulated their expression (Fig. 6 and Data S4). Two of these genes (*SlIAA4* and *SlIAA27*) were up-regulated, while the other one (*SlIAA2*) was down-regulated in *SlDof1* RNAi fruit. These data suggest that SlDof1 is involved in regulation of auxin signaling, which appears to be complex. Our results are consistent with previous work showing that Dof transcription factors could mediate auxin biosynthesis, auxin transport/perception, as well as the auxin response (Gupta et al. 2015). Interestingly, RIN was recently reported to target several *Aux/IAA* genes (Fujisawa et al. 2013; Zhong et al. 2013). This suggests that SlDof1 might cooperate with RIN in regulating these genes. RIN also regulates the auxin-responsive gene *SlSAUR69*, which plays a role in initiation of tomato fruit ripening (Shin et al. 2019). However, *SlSAUR69* was not identified as a SlDof1 target gene in our study. The function of these Aux/IAA proteins in fruit ripening requires further investigation.

In summary, we showed that SlDof1 functions as a regulator that is necessary for the normal ripening of tomato fruit. Large-scale identification of direct SlDof1 targets revealed that SlDof1 acts as both an activator and repressor during fruit ripening. SlDof1 directly regulates a set of well-known ripening-related genes, such as *ACS2*, which is involved in ethylene signaling, and *PG2A*, which is associated with cell wall metabolism. SlDof1 also targets multiple potential ripening-related genes, whose functions require further investigation (Fig. 7C). Our findings aid in the elucidation of the gene regulatory networks of fruit ripening.

## Methods

### Plant materials and growth conditions

Seeds of wild-type tomato (*S. lycopersicum* cv. Ailsa Craig) were kindly provided by Dr. James J. Giovannoni from the Boyce Thompson Institute for Plant Research, Cornell University. Wild-type and transgenic tomatoes were grown under controlled glasshouse conditions at 25 °C with a 16-h photoperiod. To determine fruit ripening stages, flowers were tagged at anthesis. Fruit samples from the wild type were harvested at the mature green (MG), breaker (Br), orange (Or), and red ripe (RR) stages, which were on average 35, 38, 41, and 44 days post-anthesis (dpa), respectively. For transgenic lines, fruit were collected at equivalent ripening stages, which were determined by the number of dpa.

### RNA isolation and quantitative RT-PCR analysis

Total RNA was extracted from 100 mg of tomato pericarp using the method of Moore et al. (2005). One microgram of total RNA was used to synthesize cDNA with the PrimeScript® RT reagent kit with gDNA Eraser (Takara). Quantitative RT-PCR was performed on a StepOne Plus Real-Time PCR System (Applied Biosystems) using SYBR Green PCR Master Mix (Applied Biosystems). The PCR reaction system and PCR conditions were set according to the manufacturer’s instructions. The cDNA was diluted 20 times and then used as the template for quantitative RT-PCR. The specific primer sequences are listed in Table S1. The primer efficiency was calculated from the slope of the standard curve (E = 10^–1/slope^), which was generated from reactions conducted in duplicate using serial dilutions of standard cDNA. A tomato *ACTIN* (Solyc11g005330) mRNA was used as the internal reference gene, and the relative expression of a specific gene was analyzed using the cycle threshold (Ct) 2^-ΔΔCt^ method. Three independent biological replicates with three technical replicates were performed for each sample.

### Phylogenetic analysis

The sequences of all 34 SlDof proteins were obtained from Cai et al. (2013). The alignment of the protein sequences was generated by ClustalX (version 2.1) software using default parameters. The phylogenetic tree was constructed by MEGA (version 6.0) using the neighbor-joining method with the Poisson correction model, pairwise deletion, and 1000 bootstrap replications (Tamura et al. 2013).

### Virus-induced gene silencing (VIGS)

The VIGS assay was performed according to the method of Quadrana et al. (2011). The specific cDNA fragments corresponding to *SlDofs* were amplified and cloned into the virus vector pTRV2 to generate pTRV2-*SlDof* constructs. A *phytoene desaturase* gene (*PDS*) fragment was inserted into pTRV2 to serve as the positive control. The pTRV2-*SlDof* and pTRV2-*PDS* constructs were then introduced into *A. tumefaciens* strain GV3101 by electroporation. For tomato plant infiltration, equivalent aliquots of *Agrobacterium* strain GV3101 containing pTRV1 or pTRV2 (empty or containing the insert) were mixed and needle-injected into inflorescence peduncles of 8-week-old Micro-tom tomato plants.

### Preparation of SlDof1 antibody and western blotting

For SlDof1-specific antibody preparation, a truncated form (amino acids 289 to 432) of *SlDof1* lacking the conserved Dof domain was amplified using primers Dof1t-F (5′-CGGGATCCTTATCATCCTCCTCTTCTTCTTC-3′) and Dof1t-R (5′-CCGCTCGAGTTACCAAGATCCTCCAGTACCAC-3′) containing *Bam*HI and *Xho*I sites, respectively. The PCR products were then cloned into the pET30a prokaryotic expression vector to produce pET30a-*Dof1*. The recombinant protein was expressed in *E. coli* BL21 (DE3) and then purified using Ni-NTA His Bind Resin according to the manufacturer’s manual (Merk). SlDof1 polyclonal antibody was prepared by immunizing rabbits with purified SlDof1 truncated protein at Beijing Protein Institute Co., Ltd. SlDof1 antibody was affinity-purified from the antisera using the AminoLink Plus Coupling Resin following the purification protocol (Thermo Scientific).

Nuclei isolation and nuclear protein extraction from pericarp of tomato fruits were performed as described previously (Wang et al. 2014). Nuclear proteins were resolved in lysis buffer (7 M urea, 2 M thiourea, 4% CHAPS, 1% dithiothreitol) and protein concentration was measured using the method of Bradford (1976). Aliquots of protein (10 μg) were separated by 12% SDS-PAGE and electro-transferred to a PVDF membrane (Millipore). Subsequent immunoblotting and protein detection were performed as described previously (Wang et al. 2014).

### Cell-free degradation assay

The cell-free degradation assay was performed following the method of Wang et al. (2009). Total proteins were extracted from tomato leaves and fruit pericarps with extraction buffer containing 25 mM Tris-HCl (pH 7.5), 10 mM NaCl, 10 mM MgCl_2_, 1 mM PMSF, 1 mM ATP, and 5 mM DTT. The protein concentration was determined following the Bradford method and the total protein extracts from leaves and pericarps were adjusted to equal concentrations (1 μg μl^− 1^) before incubation with SlDof1-HA. For generation of recombinant SlDof1-HA, full-length SlDof1 without a stop codon was amplified and inserted into the pET30a vector, using the primers Dof1-HA-F (5′-GCCATGGCTGATATCGGATCCATGGAGTCTACTCAATGGTC-3′) and Dof1-HA-R (5′-GTGGTGGTGGTGGTGCTCGAGTTAAGCGTAGTCTGGGACGTCGTATGGGTACCAAGATCCTCCAGTACC-3′), which contained the coding sequence of the hemagglutinin (HA) tag. Recombinant protein expression and purification were performed as described above, and 500 ng of purified recombinant SlDof1-HA proteins was added to 500 μl of the total protein extracts. The mixtures were incubated at 25 °C for 0, 1, 2, 3 and 4 h, and then subjected to immunoblot analysis using anti-HA antibody (Abmart). Three independent experiments were performed and the band intensity was quantified using ImageJ software (https://imagej.nih.gov/ij/index.html).

### Subcellular localization

For subcellular localization analysis, the *SlDof1* cDNA lacking the stop codon was amplified using the specific primers Dof1-eGFP-F (5′-CGGGGTACCATGGAGTCTACTCAATGGTC-3′) and Dof1-eGFP-R (5′-CGCGGATCCCCAAGATCCTCCAGTACCA-3′). The PCR product of *SlDof1* was then cloned into the pCAMBIA2300 vector containing *eGFP* at the C terminus (In-Fusion® HD Cloning Kit; Clontech). The resulting plasmid and the control (empty plasmid) were transformed into *A. tumefaciens* strain GV3101, which was subsequently infiltrated into *N. benthamiana* leaves. The eGFP fluorescence signals were observed under a confocal laser scanning microscope (Leica DM1600CS). The fluorescent dye 4′,6-diamidino-2-phenylindole (DAPI) was used for nuclear staining.

### RNAi vector construction and plant transformation

To construct the *SlDof1* RNAi plasmid, a 272-bp fragment of the *SlDof1* gene (bases 556 to 827 of the full length cDNA) was amplified using the primers Dof1-RNAi-F (5′-GCTTTATACAATTCAGGTTTTCCATTTCA-3′) and Dof1-RNAi-R (5′-CAAGATCCTCCAGTACCACTTATCATCCC-3′). The PCR product was subcloned into the PCR8/GW/TOPO Gateway entry vector (Invitrogen). The cloned fragment was then transferred into the destination vector pK7GWIWG2D by the attL × attR reaction using the LR Clonase II enzyme (Invitrogen) to generate pK7GWIWG2D*-SlDof1*.

The *SlDof1* RNAi plasmid was transformed into the *A. tumefaciens* stain GV3101 by electroporation, and *Agrobacterium*-mediated tomato transformation was performed following a previously described method (Fillatti et al. 1987). The transformed plants were selected on the basis of kanamycin resistance, and the presence of the transgene was confirmed in the T0 and T1 generation by PCR. Approximately 100 fruits from each RNAi line and the wild type were harvested for phenotypic observation.

### Ethylene production and lycopene measurement

Fruits harvested at 35, 38, 41, and 44 dpa from RNAi lines and the wild-type tomato were used for the measurement of ethylene and lycopene. Ethylene generation was determined as described previously (Wang et al. 2017). Five fruits were sealed in a jar and incubated at room temperature for 2 h. One milliliter of gas sample was taken and injected into a gas chromatograph (SQ-206, Beijing, China) equipped with an activated alumina column and a flame ionization detector. Ethylene concentrations were calculated by comparing with reagent-grade ethylene standards of known concentration and normalized by fruit weight. There were three replicates of each sample with five fruits per replicate, and the experiment was repeated twice.

Pericarp lycopene content was determined following the method of Sun et al. (2015). In brief, 5 g of tomato pericarp was homogenized with 50 ml of buffer containing a mixture of hexane-acetone-ethanol (2:1:1, v/v) in an aluminum foil-wrapped tube. The homogenate was shaken for 5 min, then 15 ml of water was added and the sample kept on ice for phase separation. The absorbance of the organic phase (hexane) at 503 nm was measured to determine the lycopene concentration. Lycopene content was calculated using the molar extinction coefficient 17.2 L mol^− 1^ m^− 1^ and expressed as μmol kg^− 1^ fresh weight. There were three replicates of each sample with five fruits per replicate, and the experiment was repeated twice.

### RNA-seq and data analysis

Total RNAs were isolated from fruit pericarp of the wild type and *SlDof1* RNAi lines at 38 dpa by the method of Moore et al. (2005). Three biological replicates were performed, and each replicate contained four to five combined fruits. The cDNA library preparation and sequencing were conducted at Beijing Genomics Institute (BGI). In brief, the mRNA was purified with oligo (dT), fragmented, reverse-transcribed into first-strand cDNA, and then synthesized into second-strand cDNA with DNA polymerase I and RNaseH. After purification, the cDNA fragments were end-repaired, poly(A)-tailed, adaptor ligated, and then PCR amplified. All cDNA libraries were sequenced on an Illumina HiSeq 2000 platform with the 100-bp pair-end sequencing strategy as described by the manufacturer (Illumina, USA). RNA-seq raw reads were filtered using SOAPnuke1.3.0 filter software to obtain clean reads, which were then mapped to the whole tomato genome (http://solgenomics.net/organism/Solanum_lycopersicum/genome) using BWA and Bowtie software. Differentially expressed genes between samples were defined by DESeq software using two separate models, based on the posterior probability of equal expression < 0.05 and false discovery rate < 0.001. GO analysis of genes with differential expression was performed using the Blast2GO software (http://www.geneontology.org), and the functional classification was performed using the BGI WEGO software (http://wego.genomics.org.cn).

### Chromatin immunoprecipitation followed by high-throughput sequencing (ChIP-seq) and data analysis

Chromatin immunoprecipitation was performed as described previously (Wang et al. 2014). Briefly, 5 g of fruit pericarp (from five plants to account for variation among individuals) was cross-linked with 1% (v/v) formaldehyde under a vacuum and ground to a powder in liquid nitrogen to isolate nuclei. The enriched nuclei were then sonicated to fragment DNA to 250–500 bp. The sheared chromatin was then incubated overnight with the affinity-purified polyclonal anti-DOF1 antibody. The chromatin before incubation with antibody was used as the input DNA control. After being captured by protein A-magnetic beads (Millipore), the protein-chromatin immunocomplexes were washed, eluted, and reverse cross-linked. The immunoprecipitated DNA and input DNA were then purified with phenol/chloroform extraction and pooled separately.

ChIP-seq libraries were constructed using the Paired-End DNA Sample Prep kit (Illumina) and sequenced at BGI with the Illumina HiSeq 2000 platform. The raw reads were filtered, and sequence reads with low quality bases, adaptors, or contamination were eliminated. After filtering, clean reads were mapped to the tomato genome (http://solgenomics.net/organism/Solanum_lycopersicum/genome) using the SOAPaligner/SOAP2 software, allowing for up to two mismatches and no gaps. Uniquely mapped reads were submitted to MACS software (Version 1.4.2) to identify the enriched peaks with default settings using input DNA as the background. The peak calling was performed separately for the three biological replicates, and the overlapping peaks among the three biological replicates were selected as candidates for further analysis. Data were visualized using the UCSC Genome Browser (http://genome.ucsc.edu/). The genomic distribution of SlDof1 binding peaks relative to gene structure was determined by calculating the frequency of binding peaks in the following regions: (1) gene body, i.e., from the transcription start site to the translation termination site, (2) gene promoter region, i.e., 2 kb upstream of the transcription start site, (3) downstream region, i.e., from the translation termination site to 2 kb downstream, and (4) intergenic region. If a peak was located within 2 kb upstream of a gene and 2 kb downstream of another gene, the peak was counted as being in both regions. Peaks existing within 2 kb upstream of two different genes were counted twice. Motif enrichment analysis among SlDof1 binding peaks was performed using Discriminative Regular Expression Motif Elicitation (DREME) software (Bailey, 2011). Kyoto Encyclopedia of Genes and Genomes (KEGG) pathway enrichment analysis was conducted using Fisher’s exact test with Bonferroni correction (Kanehisa and Goto, 2000).

### ChIP-quantitative PCR (ChIP-qPCR)

For ChIP-qPCR analysis, the isolated ChIP-DNA samples were used as template for real-time PCR amplification with specific primers (Table S2). The data are presented as the percentage of DNA fragments coimmunoprecipitated with specific (anti-SlDof1) or non-specific (preimmune IgG) antibodies relative to the input DNA.

### Transcriptional activity assay

For transcriptional activity analysis in *N. benthamiana* leaves, a dual luciferase reporter system was used according to Feng et al. (2016) with some modification. The promoters (2 kb upstream from the transcription start site) of putative target genes were amplified using primers listed in Table S3 and cloned into the pGreen0800II dual reporter vector upstream of the *firefly luciferase* (*LUC*) reporter gene, with a *renilla luciferase* (*REN*) reporter gene under the control of the CaMV35S promoter as an internal control. The coding sequence of *SlDof1* was cloned into the effector plasmid pCAMBIA2300 with expression driven by the CaMV35S promoter using primers Dof1-Spel-F (5′-CCCACGCGTTACGTAACTAGTATGGAGTCTACTCAATGGT-3′) and Dof1-Spel-R (5′-GACTCTAGAGAGCTCACTAGTCCAAGATCCTCCAGTACC-3′). The empty pCAMBIA2300 plasmid was used as a negative control. The reporters and effectors were co-transformed into *N. benthamiana* leaves using *A. tumefaciens* strain GV3101. After infiltration, plants were incubated at 22 °C for 48 h before analysis. LUC and REN luciferase activities were detected with a dual-luciferase assay kit (Promega), and the transcriptional activity was reported as the ratio of LUC to REN.

### Supplementary Information

**Additional file 1: Figure S1.** Expression analysis of the potential off-targets of the RNAi construct. **Figure S2.** Overview of RNA-seq data from three biological replicates. **Figure S3.** Overview of ChIP-seq data from three biological replicates. **Figure S4.** ChIP-qPCR assay reveals that *PSY1* and *ADH2* are not direct targets of SlDof1. **Figure S5.** *SlDof1* is not a direct target of RIN.

**Additional file 2: Table S1.** Primer sequences used for quantitative RT-PCR analysis. **Table S2.** Primer sequences used for ChIP-qPCR analysis. **Table S3.** Primer sequences of promoters used in transcriptional activity assays.

**Additional file 3: Data S1.** Differentially expressed genes in *SlDof1*-silenced fruit revealed by RNA-seq analysis.

**Additional file 4: Data S2.** SlDof1 binding peaks in three biological replicates of a ChIP-seq assay.

**Additional file 5: Data S3.** SlDof1 binding peaks in promoter regions and the associated genes in a ChIP-seq assay.

**Additional file 6: Data S4.** Overlap between genes identified in RNA-seq and ChIP-seq analyses.

## Data Availability

Sequence data from this article can be found in the Sol Genomics Network. RNA-seq and ChIP-seq data have been deposited in the Gene Expression Omnibus (GEO) database under accession numbers GSE119493 and GSE119492, respectively.
