# Supplementary material for: Genome-wide binding analysis of the tomato transcription factor SlDof1 reveals its regulatory impacts on fruit ripening
Source: Mol Hortic. 2021 Aug 28;1:9. doi: 10.1186/s43897-021-00011-y (PMC10514982; doi:10.1186/s43897-021-00011-y)
Supplement: Supplementary file 1 — Additional file 1: Figure S1. Expression analysis of the potential off-targets of the RNAi construct. Figure S2. Overview of RNA-seq data from three biological replicates. Figure S3. Overview of ChIP-seq data from three biological replicates. Figure S4. ChIP-qPCR assay reveals that PSY1 and ADH2 are not direct targets of SlDof1. Figure S5. SlDof1 is not a direct target of RIN. [file 43897_2021_11_MOESM1_ESM.pdf]

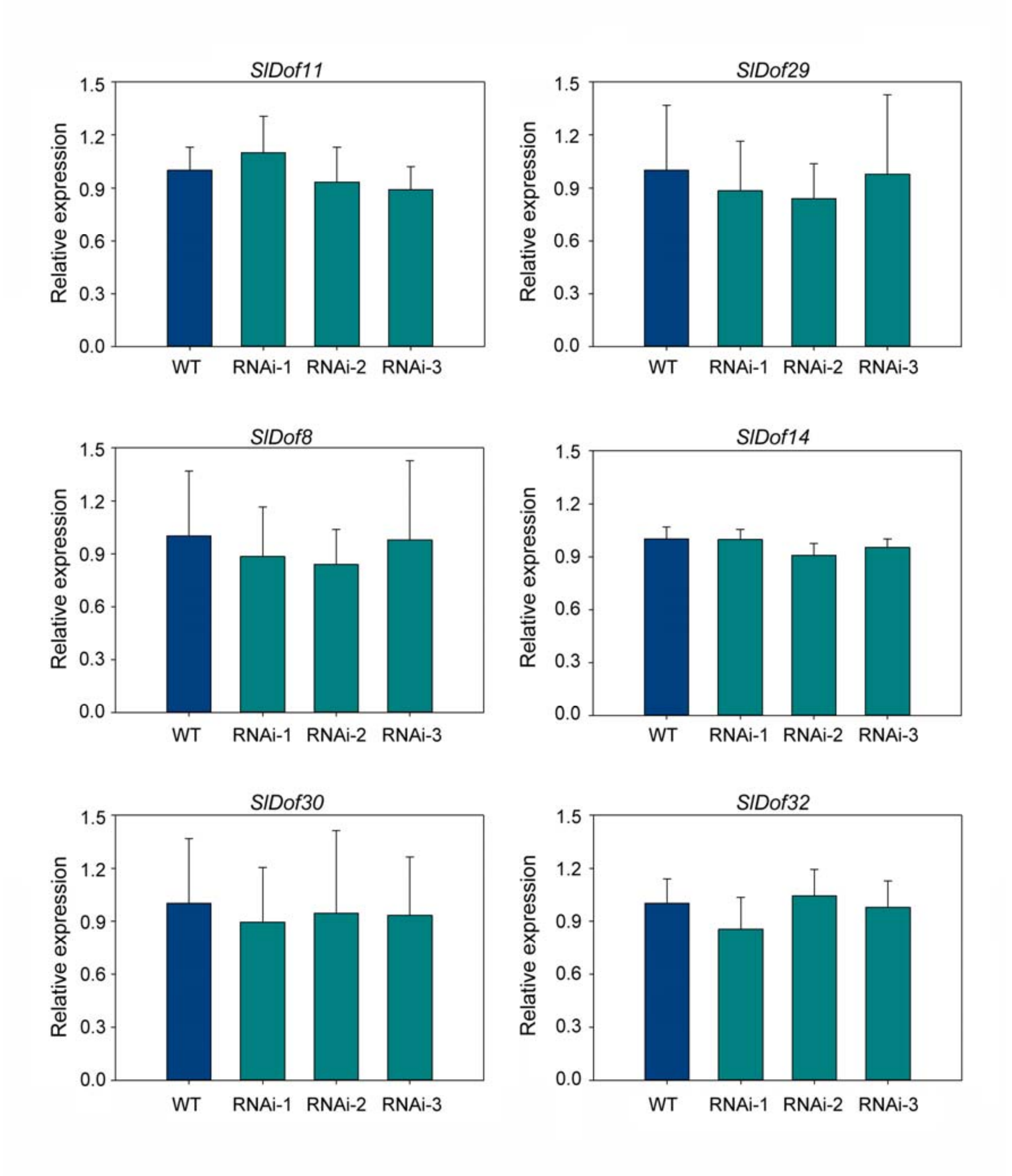

Figure S1. Expression analysis of the potential off-targets of the RNAi construct

Total RNA was isolated from leaves of the wild-type (WT) and *SlDof1* RNAi lines (RNAi-1, RNAi-2, and RNAi-3) and submitted to quantitative RT-PCR analysis. The gene expression levels were normalized against the *ACTIN* gene, followed by normalization against the expression in the WT. Values are expressed as the means ± SD of three independent experiments.

A

Sample name	Clean reads	Reads aligned	Gene map Rate
WT-Rep.1	48,853,752	36,216,348	73.88%
WT-Rep.2	50,649,908	38,579,260	76.17%
WT-Rep.3	50,614,290	39,182,164	77.41%
SlDof1-RNAi-Rep.1	50,633,086	38,666,844	76.37%
SlDof1-RNAi-Rep.2	48,866,992	36,319,220	74.16%
SlDof1-RNAi-Rep.3	50,642,098	38,892,716	76.80%

B

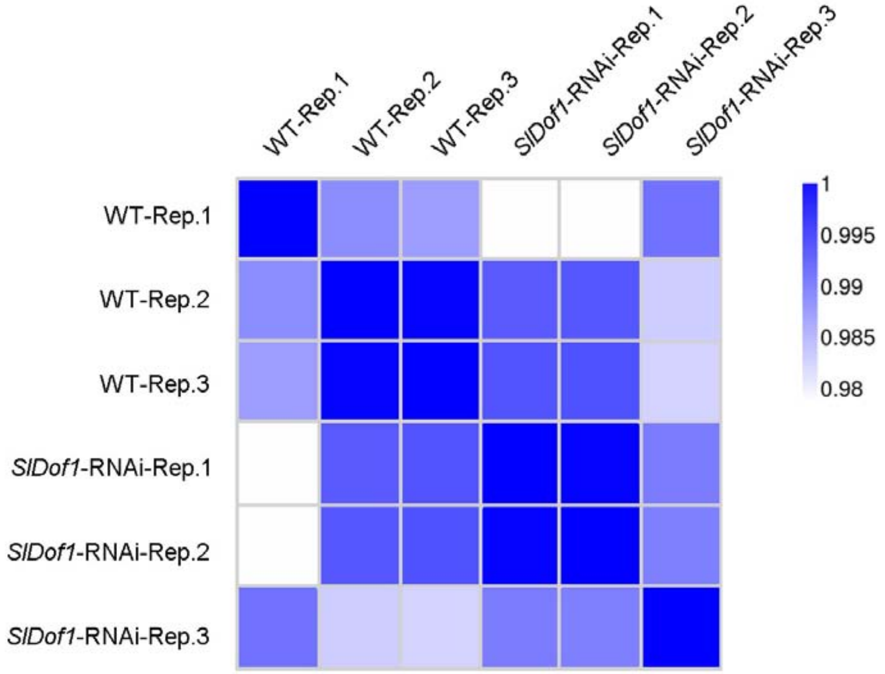

Figure S2. Overview of RNA-seq data from three biological replicates

(**A**) Numbers of clean reads, aligned reads, and the percentage of aligned reads in the wild-type (WT) and *SlDof1* RNAi fruit. (**B**) The correlation analysis of RNA-seq data. The matrix color changes from blue to white gradually, representing the correlation from high to low. Rep.1, replicate 1; Rep.2, replicate 2; Rep.3, replicate 3.

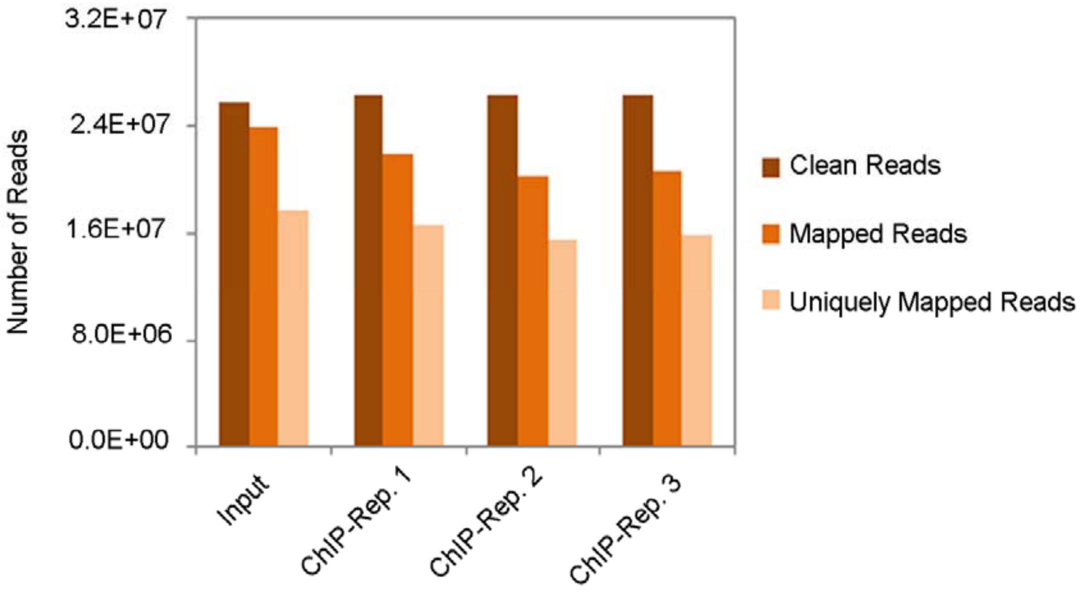

Figure S3. Overview of ChIP-seq data from three biological replicates

Numbers of clean reads, mapped reads, and uniquely mapped reads in each sample are shown. The value was expressed in exponential notation, replacing part of the number with E+n, where E multiplies the preceding number by 10 to the n*th* power. Rep.1, replicate 1; Rep.2, replicate 2; Rep.3, replicate 3.

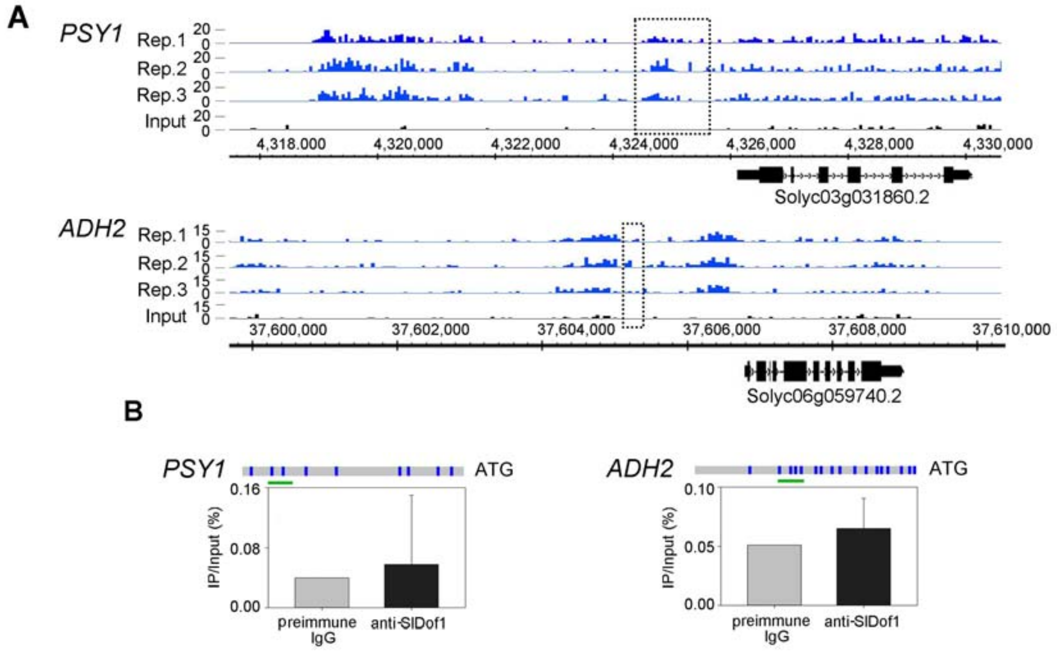

Figure S4. ChIP-qPCR assay reveals *PSY1* and *ADH2* are not direct targets of SlDof1

(**A**) The raw chromatin immunoprecipitation sequencing peaks for *PSY1* and *ADH2* from three biological replicates. Black dot-line rectangles mark the peak regions that were detected in all three biological replicates within 2 kb upstream of the transcription start site. The structure of the corresponding gene is presented below with black bars representing exons and lines representing introns. The direction of transcription is indicated by arrows. (**B**) ChIP-qPCR analysis reveals SlDof1 does not bind to the promoter region of *PSY1* or *ADH2*. The promoter structures of *PSY1* and *ADH2* are presented. Blue boxes represent the binding motifs of SlDof1. Green lines indicate the position used for ChIP-qPCR. All primers were designed based on the peak sequences enriched in chromatin immunoprecipitation sequencing. Values are the percentage of DNA fragments that co-immunoprecipitated with anti-SlDof1 antibodies or non-specific antibodies (preimmune IgG) relative to the input DNAs. Error bars represent the SD of three independent experiments.

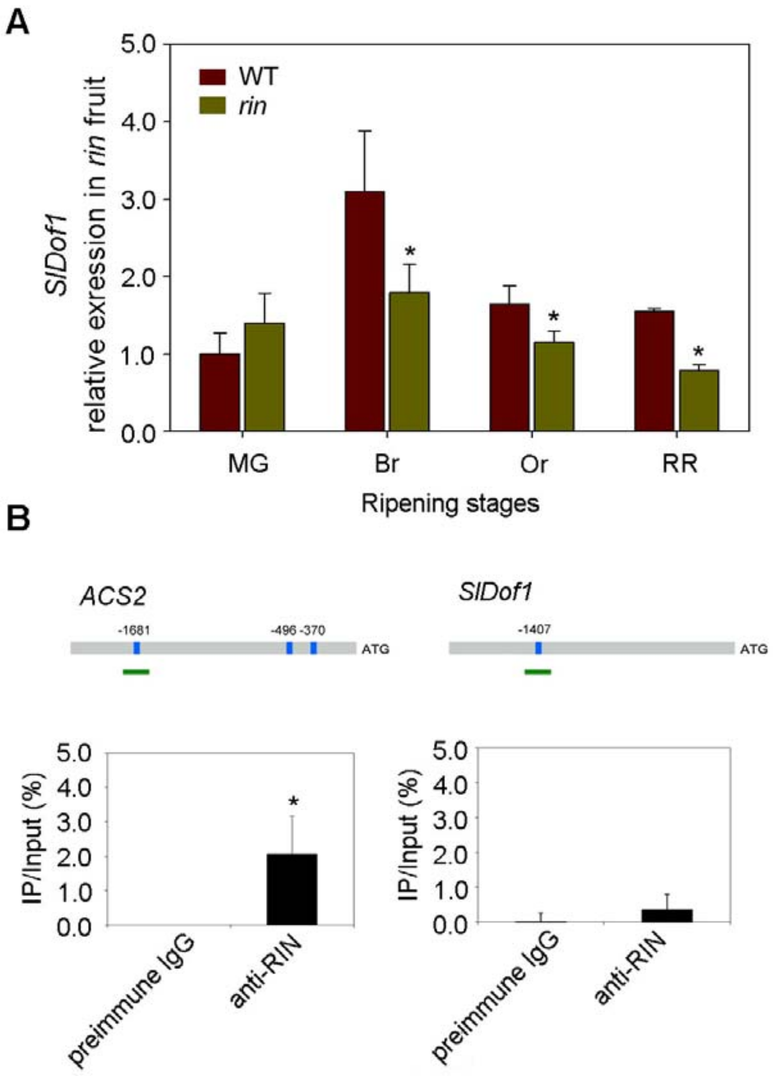

Figure S5. *SlDof1* is not a direct target of RIN

(**A**) The mRNA levels were measured by quantitative RT-PCR. Total RNA was isolated from fruits of the wild type (WT) and *rin* mutant. The stages of fruit ripening include mature green (MG), breaker (Br), orange (Or), and red ripe (RR). The *ACTIN* gene was used as an internal control. Values are expressed as the means ± SD of three independent experiments. (**B**) ChIP-qPCR analysis reveals RIN does not bind to promoter region of *SlDof1*. The promoter structures of *SlDof1* is presented. Blue boxes represent the binding motifs of RIN. Green lines indicate the position used for ChIP-qPCR. The binding of RIN protein to the promoter of *ACS2*, a known RIN-target gene was used as a positive control. Values are the percentage of DNA fragments that co-immunoprecipitated with anti-RIN antibodies or non-specific antibodies (preimmune IgG) relative to the input DNAs. Error bars represent the SD of three independent experiments. Asterisks indicate significant differences ($P < 0.05$; Student's *t*-test).
