# Supplementary material for: Genome-wide binding analysis of the tomato transcription factor SlDof1 reveals its regulatory impacts on fruit ripening
Source: Mol Hortic. 2021 Aug 28;1:9. doi: 10.1186/s43897-021-00011-y (PMC10514982; doi:10.1186/s43897-021-00011-y)
Supplement: Supplementary file 2 — Additional file 2: Table S1. Primer sequences used for quantitative RT-PCR analysis. Table S2. Primer sequences used for ChIP-qPCR analysis. Table S3. Primer sequences of promoters used in transcriptional activity assays. [file 43897_2021_11_MOESM2_ESM.pdf]

Table S1. **Primer sequences used for quantitative RT-PCR analysis.**

Gene	ITAG gene ID[a]	Primers	DNA sequences (5'-3')
SlDof1	Solyc01g096120	Forward	GGGATTACACTGGGTTGAGTATGTT
		Reverse	GCTAGCACAGAAACCCTAGAAGCT
SlDof2	Solyc02g065290	Forward	TTGCCCACGCTGTGACTCTATC
		Reverse	TGGCGTGGCTGAGACAAATTGTAG
SlDof3	Solyc02g067230	Forward	CAGGAGAGGCTGCAAAGAGTTC
		Reverse	ACTTCCACCAACTGAATCAACGG
SlDof4	Solyc02g076850	Forward	TGGTGGCTCATGAAGATCAATGGC
		Reverse	AGGTGCTTCTCCTCCTCTTAGCTG
SlDof5	Solyc02g077950	Forward	CAGCAAATGAAGGATCCGAACAAC
		Reverse	AGCTGACATGATCACTCCGATCC
SlDof6	Solyc02g077960	Forward	AGGTGGAACCCTAAGGAACGTC
		Reverse	CCGCCCTTCATTGTGCGTTTAC
SlDof7	Solyc02g078620	Forward	CACAATACTCGACCGGAACCAC
		Reverse	CACCTTGGACATTTCAACGACTCC
SlDof8	Solyc02g088070	Forward	CACCACTATGAATGGAGCTTCACC
		Reverse	AGAAGGCCAACAGGAAGAAAGGG
SlDof9	Solyc02g090220	Forward	TGCGAAACGGTCCCGTACAATC
		Reverse	AGAGAGCGTGGAGGACAAACAG
SlDof10	Solyc02g090310	Forward	TTTGGATTCGGCTCCGGTAACTG
		Reverse	TGTTGAAAGGGTCGGGTTCCAG
SlDof11	Solyc03g082840	Forward	ACAGATCTGGCGAACCCTAACG
		Reverse	AGTACCACCACTGGGTATTGGG
SlDof12	Solyc03g112930	Forward	CATGACGATCAATTAGGCAGCAAC
		Reverse	AGATCCTAGGCCACCATTACCAG
SlDof13	Solyc03g115940	Forward	CATACGCTGCTGTTCCTTTCCC
		Reverse	ATAAGGAGGAGGGCAGAATGGC
SlDof14	Solyc03g121400	Forward	CCCTGCAACTCCTCCATTAACATC
		Reverse	TGCAGGTATACCGAATGGCTCTG

SlDof15	Solyc04g070960	Forward	TTGGCTATGCAGTCGGTTGGAC
		Reverse	TGCACTCTTTGGCACTCGTTTG
SlDof16	Solyc04g079570	Forward	TGGGTGGGTTTGTGCCATTAGG
		Reverse	TGGGCCAATCAAGATTCCCAAGC
SlDof17	Solyc05g007880	Forward	CCTCGTTACTTCTGCAAGAACTGC
		Reverse	ACCAGATCCCACAGGCACATTTC
SlDof18	Solyc05g054510	Forward	CCCAATTGGCATCATGAGCAAGG
		Reverse	CTAGTTGAGCCAACTGGACCAC
SlDof19	Solyc06g005130	Forward	TCTCGTGCTGGCTTTCTTGATAC
		Reverse	TGGTGCAAGAAGCCATTTGATGC
SlDof20	Solyc06g062520	Forward	TTCCAGTTGGTGGAAGCTCTCG
		Reverse	TCGTCATCGTTGAGGAGCGTTTAC
SlDof22	Solyc06g069760	Forward	ACTCCAGCGGGAAACTTCATGG
		Reverse	GCTCTGTAAGTGATCTGCGCACTG
SlDof23	Solyc06g071480	Forward	TGGTACTACTGGTCTGATCAACGG
		Reverse	AGCAATAATGTGGTCCGATTCTCG
SlDof24	Solyc06g075370	Forward	TCCCTCTAGTGCACAGCATCAG
		Reverse	TGGCTTTGTACTCACCAACATGC
SlDof27	Solyc08g082910	Forward	TGCTAACAGCCCAACACTTTCTC
		Reverse	ACACATGACATGGTAGACCCAAAC
SlDof28	Solyc09g010680	Forward	ACCTTGGTGGTGAGAGTACGAG
		Reverse	TCACGGAGGCCATTTGATTTAGC
SlDof29	Solyc10g009360	Forward	CATGGTGAATAAGGAATTGAAAGAAA
		Reverse	TCAAAGAGTAAAGTGAATAACAAGGAAACT
SlDof30	Solyc10g086440	Forward	TGTGGGTGGTGGAGGTTCAATC
		Reverse	TTTGCTAACCGGGCTCGATCTG
SlDof32	Solyc11g066050	Forward	CCAACCGCGCCATTTCTGTAAG
		Reverse	TCCCACCGGCACATTTCTCAAG
SlDof33	Solyc11g072500	Forward	TCAGCAAAGCGATCAAGCCAAG
		Reverse	TGGCGAATGCTAGGCTCAAATCAG

NOR	Solyc10g006880	Forward	AGAGAACGATGCATGGAGGTTTGT
		Reverse	ACTGGCTCAGGAAATTGGCAATGG
FUL1	Solyc11g005330	Forward	GTTTTGCCACAACAACTGGACTC
		Reverse	CTTGCTGCTGTGAAGAACTACC
ACS2	Solyc01g095080	Forward	AATGTCAAGAGCCAGGGTGGTTCC
		Reverse	TCCTCGCGAGCGCAATATCAAC
Nr	Solyc09g075440	Forward	TGTTCCCTATGCCAAGTGATGGC
		Reverse	ACACCCAGTATCTCTAACCTGGAC
PSY1	Solyc03g044300	Forward	ATCAATAGAGGTGGTGGAAAGCAA
		Reverse	CCAGATGGAGTAGCCAAAATAGCA
PDS	Solyc03g123760	Forward	TTGACAGAAAACTGAAGAACACATA
		Reverse	TGCAAAAACCAATTCCAACATAGAC
PG2A	Solyc10g080210	Forward	TGGACAAGTATGGTGGCCAAGTTC
		Reverse	TCCAGAAGGTTAAGGCCGTTGG
PME1	Solyc07g064170	Forward	TCCCGCTAAAGCTATGCCGTTC
		Reverse	TTATGCTTGCTCTGCCCTGCTC
LoxC	Solyc01g006540	Forward	CGGTGGTGCCGGCATTGGAA
		Reverse	CGGTGGTGCCGGCATTGGAA
ADH2	Solyc06g059740	Forward	ACACCGTGGTTCATGTTGGATGTG
		Reverse	TCAAACTTGCTCCAAGGCCTGTC
ACTIN	Solyc11g005330	Forward	TGTCCCTATTTACGAGGGTTATGC
		Reverse	AGTTAAATCACGACCAGCAAGAT

[a]ITAG, the International Tomato Annotation Group release version 2.5.

Table S2. Primer sequences used for ChIP-qPCR analysis.

Gene	ITAG gene ID[a]	Position	Primers	DNA sequences (5'-3')
ACS2	Solyc01g095080	-869 ~	Forward	TTGTAATGGCAGTCTCTTTTTCTC
		-718	Reverse	CCGGGTTTTCACTTCATCAC
NR	Solyc09g075440	-270 ~	Forward	GCCTGTAAGTAACCATACCTCTTAA
		-118	Reverse	GGCATGTAGCATTTTCATCCAA
PG2A	Solyc10g080210	-1023 ~	Forward	GAATCGAAGCACCAATCTAATTT
		-848	Reverse	ATTCCTTCGTCTCAATTATGTGATG
NOR	Solyc10g006880	-1533 ~	Forward	CATATGAGCGCCACGAATTC
		-1383	Reverse	ACGCATGCAAGTTGGTACCA
MC	Solyc05g056620	-1882 ~	Forward	GCGCCCTTTGTTATTACTTTTCCT
		-1707	Reverse	GCGCCACACATTTTAGTATTCCTTA
SlIAA2	Solyc06g084070	-1416 ~	Forward	AAAAGCTAAGCGATTTAACAAGTG
		-1215	Reverse	TATTGCAGTGGACTGACTAAAGC
SlIAA4	Solyc06g053840	-620 ~	Forward	GCCCCACCCCTAAATAGTAGT
		-382	Reverse	CCCCAAAACTTACAAATCACAA
SlIAA27	Solyc03g120500	-1218 ~	Forward	CCTATTTGATGGTTGGCACTAA
		-963	Reverse	CGTTGCACTGAGTTTGTCTAATAC
SlREV8	Solyc10g084370	-321 ~	Forward	TAGAGCATGTGCATCTACGTATTC
		-121	Reverse	CAAGTGGTCTACGCCGATTT
SlDIV2	Solyc06g076770	-328 ~	Forward	GTCATAAAGGGAAAAAAGGAAGAA
		-128	Reverse	TCCACTGTTTTAGTCCTTCAAAT
PSY1	Solyc03g031860	-1660 ~	Forward	GCTAAATCGAAAATTGAATCGTT
		-1407	Reverse	TTCCACTGTTTTAGTCCTTCAAAT
ADH2	Solyc06g059740	-1597 ~	Forward	CTTCAAGACTCTAATTTTTTTTA
		-1386	Reverse	AAAAAGTAGTTATCAATATTAGGAA
SlDof1	Solyc01g096120	-1491~	Forward	AGTAAAGTGAAACACTGAAAAGTCTA
		-1362	Reverse	TGTAACATGTTCTAATTGTCCTCTAT
ACS2	Solyc01g095080	-1707~	Forward	TCACAAACGAGCTATTCTAAAAA
		-1605	Reverse	CCTTACATCATTTATTATTACAA

[a]ITAG, the International Tomato Annotation Group release version 2.5.

Table S3. Primer sequences of promoters in transcriptional activity assay.

Gene	ITAG gene ID[a]	Primers	DNA sequences (5'-3')
ACS2	Solyc01g095080	Forward	GCCCATGATAAGCCTCCAT
		Reverse	TTTTTTTTTACTAAATGAGTTTAGAAGTGAAGAAAT
PG2A	Solyc10g080210	Forward	TTTGGGGCCTAAGACAAAGG
		Reverse	GATATATTGTTATATGGTATGGTTTTTAAACTTGT
SlIAA2	Solyc06g084070	Forward	TTATTGTTTGGTTGTGGGCG
		Reverse	TTCTTATAATTTCACGGTGAAAGCTGAA
SlIAA4	Solyc06g053840	Forward	AATGTTTGCTATATGTGAAAGTTGCC
		Reverse	TATAGAGGGTCCACCCGTT
SlIAA27	Solyc03g120500	Forward	ACATATAAAAAGAGATGGATGATGTTGGT
		Reverse	AGACTTCTTTTGGTGCTTTTGTAGA

[a]ITAG, the International Tomato Annotation Group release version 2.5.
